# Phylogenetic-informed graph deep learning to classify dynamic transmission clusters in infectious disease epidemics

**DOI:** 10.1093/bioadv/vbae158

**Published:** 2024-11-07

**Authors:** Chaoyue Sun, Yanjun Li, Simone Marini, Alberto Riva, Dapeng Oliver Wu, Ruogu Fang, Marco Salemi, Brittany Rife Magalis

**Affiliations:** NSF Center for Big Learning, University of Florida, Gainesville, FL 32611, United States; Department of Electrical and Computer Engineering, Herbert Wertheim College of Engineering, University of Florida, Gainesville, FL 32603, United States; NSF Center for Big Learning, University of Florida, Gainesville, FL 32611, United States; Department of Medicinal Chemistry, Center for Natural Products, Drug Discovery and Development, University of Florida, Gainesville, FL 32610, United States; Department of Epidemiology, University of Florida, Gainesville, FL 32610, United States; Interdisciplinary Center for Biotechnology Research, University of Florida, Gainesville, FL 32610, United States; NSF Center for Big Learning, University of Florida, Gainesville, FL 32611, United States; Department of Electrical and Computer Engineering, Herbert Wertheim College of Engineering, University of Florida, Gainesville, FL 32603, United States; J. Crayton Pruitt Family Department of Biomedical Engineering, Herbert Wertheim College of Engineering, University of Florida, Gainesville, FL 32611, United States; Department of Pathology, Immunology, and Laboratory Medicine, University of Florida, Gainesville, FL 32610, United States; Emerging Pathogens Institute, University of Florida, Gainesville, FL 32610, United States; Department of Biochemistry and Molecular Genetics, University of Louisville, Louisville, KY 40202, United States

## Abstract

**Motivation:**

In the midst of an outbreak, identification of groups of individuals that represent risk for transmission of the pathogen under investigation is critical to public health efforts. Dynamic transmission patterns within these clusters, whether it be the result of changes at the level of the virus (e.g. infectivity) or host (e.g. vaccination), are critical in strategizing public health interventions, particularly when resources are limited. Phylogenetic trees are widely used not only in the detection of transmission clusters, but the topological shape of the branches within can be useful sources of information regarding the dynamics of the represented population.

**Results:**

We evaluated the limitation of existing tree shape metrics when dealing with dynamic transmission clusters and propose instead a phylogeny-based deep learning system –*DeepDynaTree*– for dynamic classification. Comprehensive experiments carried out on a variety of simulated epidemic growth models and HIV epidemic data indicate that this graph deep learning approach is effective, robust, and informative for cluster dynamic prediction. Our results confirm that *DeepDynaTree* is a promising tool for transmission cluster characterization that can be modified to address the existing limitations and deficiencies in knowledge regarding the dynamics of transmission trajectories for groups at risk of pathogen infection.

**Availability and implementation:**

*DeepDynaTree* is available under an MIT Licence in https://github.com/salemilab/DeepDynaTree.

## 1 Introduction

Molecular epidemiology has become increasingly standard in pathogen outbreak surveillance and modeling of epidemic spread. Owing to short generation times and/or infidelity of pathogen replication machinery, the accumulation of mutations in viruses and bacteria is relatively rapid (compared to eukaryotes), occurring on a similar timescale as epidemiological processes (Grenfell *et al.* 2004, Volz *et al.* 2013). Genomic data can thus act as a fingerprint for specific events, such as rapid growth in an infected population following the introduction of a new variant or infection decline as a result of vaccine implementation (Zhu *et al.* 2022). Specifically, phylogenetic trees, reconstructed from the set of mutational information at genomic sites, describe relationships between individual samples, represented by branch lengths and branching patterns. Branches throughout the tree can adopt generalized shapes, or topological features, that can provide critical information as to the underlying contact dynamic (Leventhal *et al.* 2012, Böhme *et al.* 2018) and population dynamics over time (Grenfell *et al.* 2004).

In the example of a localized community outbreak, transmission is inherently dynamic over time, typically characterized by early, unchecked growth followed by a natural decline owing to the decrease in number of susceptible individuals (i.e. all individuals are ultimately either infected or immune). The branching patterns within a tree typifying this dynamic are used by Barido-Sottani *et al.* (2018) to identify transmission clusters and estimate relevant epidemiological parameters. However, transmission dynamics over time can present in a variety of other flavors, even among community-based clusters, owing to factors such as limited temporal sampling or change in the susceptible fraction of the population. In making the assumption that the number of susceptible individuals is already beginning to decline, one is restricted to *post hoc* outbreak analysis, whereas the identification of clusters of transmission is most critical during the early stages of an outbreak—prior to the observance of this decline and during which design of a more targeted use of often limited public health resources is necessary.

Despite the importance of characterization of dynamic behavior among transmission clusters, no method currently exists to perform this task. There exist metrics to describe topological tree shape over an entire phylogeny (e.g. Pybus and Harvey 2000, Frost and Volz 2013, Volz and Didelot 2018) or even identify recent rapid spread among sub-trees (e.g. Oster *et al.* 2018), and these methods are covered in more depth in the Supplementary Section SA); however, we report that these methods, even in combination, fail to accurately classify dynamic transmission clusters within an outbreak phylogeny. To address the limitations of existing models, we propose *DeepDynaTree*, a method based on graph neural networks (GNNs) principles that are capable of learning both pre-calculated tree shape metrics and phylogenetic tree structure to accurately predict the dynamics of transmission clusters within a phylogeny. The GNN approach has been successfully applied in increasing domains wherein the data are represented as a graph structure connected via complex relationships. Few efforts have been made, however, to exploit the GNN’s capacity to learn from the phylogenetic tree as an existing graph, where neighboring individuals are expected to have a similar transmission pattern. Another unique contribution of this method is an expanded message-passing mechanism from nodes within the tree to edges (i.e. branches) using the dual graph system, whose nodes and edges correspond to those of the original phylogenetic tree. Long Short-Term Memory (LSTM) (Hochreiter and Schmidhuber 1997) model, equipped with the chain-like network structure, is well-suited to extract the temporal sequence information and shows its success in a series of graph learning tasks like skeleton-based action recognition (Si *et al.* 2019), network link prediction (Chen *et al.* 2022), and traffic forecasting (Bogaerts *et al.* 2020). In this study, we utilized two LSTM models for the primal and dual graphs accordingly to capture the sequential pattern generated across rounds of message-passing iterations.

We thus propose a Primal-Dual Graph LSTM (*PDGLSTM*) model, which alternates to pass the learned node/edge representation information between the primal and dual graphs. Using simulated early outbreaks with high-risk dynamic clusters, we demonstrate herein the effectiveness of our method and provide detailed interpretation of the model predictions.

## 2 Materials and methods

### 2.1 Computational workflow

**Figure 1.**
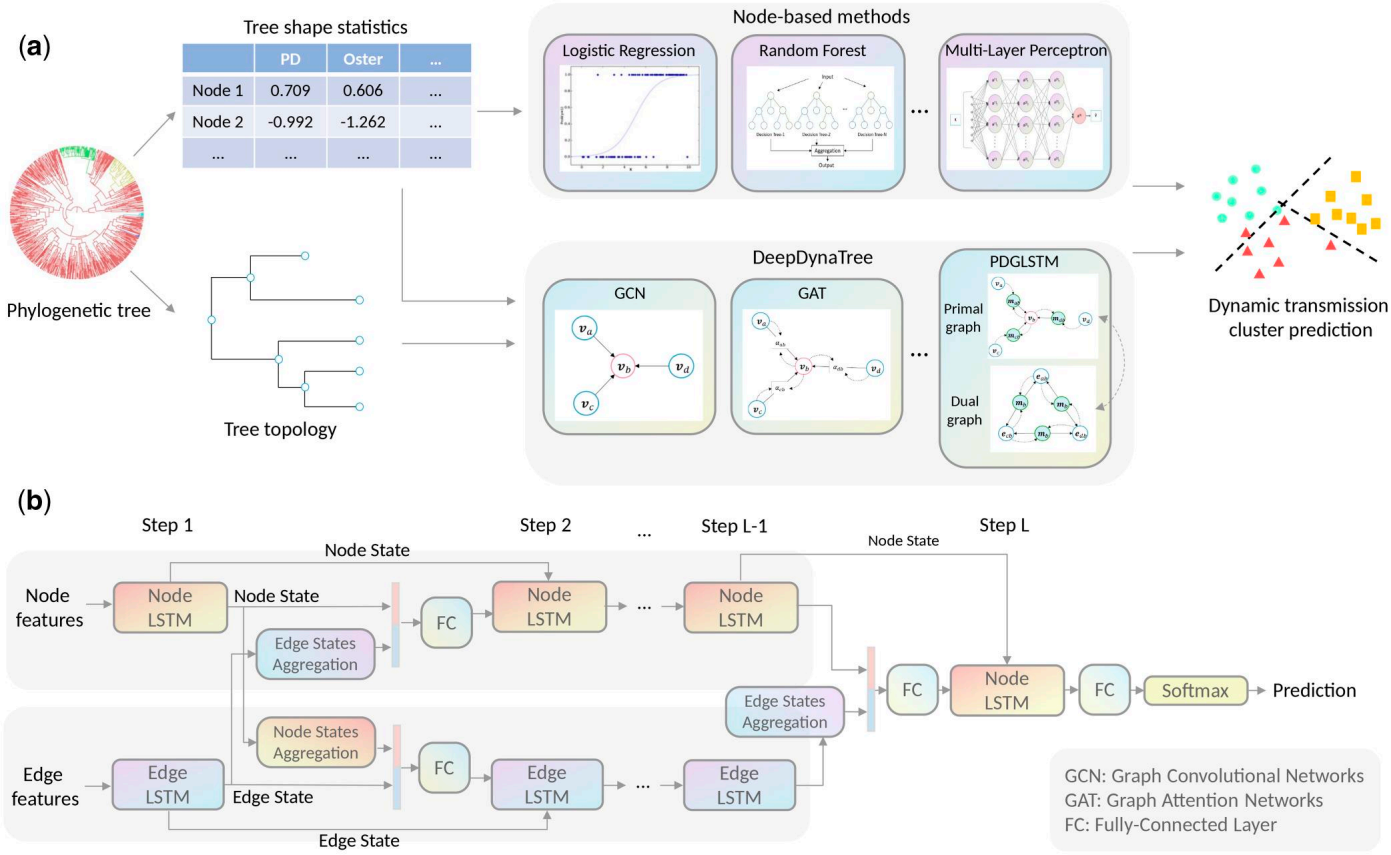
Schematic of dynamic transmission cluster classification workflow and our proposed *DeepDynaTree-PDGLSTM* architecture. (a) Data simulation and model prediction. Agent-based simulation of early-midway epidemic outbreaks involving acute and chronic respiratory infections was performed to create complex epidemiological networks, focusing on dynamic clusters of transmission. Infection networks were translated to strictly bifurcating, phylogenetic trees. Conventional node-based methods, utilizing information in the form of tree shape metrics, were compared to our proposed GNN-based *DeepDynaTree* method, capable of learning both the metrics and tree topological structure, for transmission dynamic classification. We compared different GNN variants within *DeepDynaTree*, in addition to proposing a new variant referred to as the Primal-Dual Graph Long Short-Term Memory (*PDGLSTM*) model. (b) The architecture of our proposed *PDGLSTM*. Two parallel LSTM models take the node and edge features as the initial inputs and produce a set of hidden states. For node feature updating, an edge state aggregation module is applied to aggregate the messages from the inbound edges, then the concatenated representation (including the edge messages and the last node features) is passed to the Node-LSTM model to generate the new node state. A similar strategy is also used for edge feature updates. After several rounds of node and edge feature updating and communication, the hidden state of the Node-LSTM is used to predict the corresponding node’s cluster transmission type.

The overarching goal of this study was to successfully predict the dynamics of transmission clusters based on a subset of previously developed, generalized tree shape metrics, as well as the underlying phylogenetic tree shape commonly derived from genomic data. Figure 1a illustrates the entire computational workflow for dynamic transmission cluster prediction. Briefly, simulations of early-midway epidemic outbreaks were performed using the *nosoi* (Lequime *et al.* 2020) agent-based stochastic simulation platform, which is designed to take into account the influence of multiple variables on the transmission process (e.g. population structure and dynamics) to create complex epidemiological simulations. The resulting transmission network was translated in *nosoi* to a strictly bifurcating tree, representative of the underlying pathogen evolution, and used as ground truth in the training and evaluation of the prediction algorithms. Tree nodes were categorized as belonging to transmission among the background (i.e. majority) infected population or to one of three to seven risk groups comprising clusters of transmission. Risk group nodes were classified into three main categories—static, growing, or decaying—based on pre-defined transmission dynamics in the simulation (Supplementary Table S3), and the three categories served as the classification labels during modeling. Various metrics describing the topological tree shape were used to represent each risk group node, wherein topological information was represented by branching patterns and lengths. Metrics included Pybus’s γ (Pybus *et al.* 2000), Oster statistic (Oster *et al.* 2018), phylogenetic diversity, basic reproductive number ($R_{0}$), average and maximum growth rates for estimated effective population size ($N_{e}$), fraction of time spent in growth rate, shape of lineages through time, number of “cherries,” and branch length difference (see Supplementary Section SA). We grouped the cluster prediction methods into two categories according to input information for model development (1) node-based methods, in which each cluster was treated individually and solely utilized the node features (i.e. the generalized tree shape metrics); (2) *DeepDynaTree*, wherein a GNN was leveraged to encode a phylogenetic tree as an existing bi-directed graph and directly learned from its structure using a message-passing mechanism. We also went one step further to propose a novel variant of GNNs, referred to as *PDGLSTM* (Fig. 1b), which enables the incorporation of the phylogenetic topological data and sequential processing of tree shape descriptors during nodes-edges communications.

### 2.2 Dynamic transmission cluster simulation

Simulation of early-midway epidemic outbreaks was performed using the *nosoi* (Lequime *et al.* 2020) agent-based stochastic simulation platform. Beginning with a single infected individual, rate of transmission of infection to susceptible individuals varied according to two scenarios (1) transmission of an acute infectious respiratory virus (ARI) or (2) transmission of *Mycobacterium tuberculosis* (TB) among populations of HIV-infected or HIV-uninfected individuals, as described in (Goldstein *et al.* 2022). Detailed simulation setup, including transmission parameters, is described in Supplementary Section SD. For each of the ARI and TB scenarios, 10 000 simulations beginning with a single infected individual were performed, resulting in 8574 and 7029 successful outbreaks, respectively. Overall, 15 603 phylogenetic trees included 55 037 093 nodes and 55 021 490 edges. Tree nodes (i.e. individuals) were categorized into two types—nodes belonging to transmission among the background, or majority, infected population (48 083 516 nodes in total), and the other belonging to one of seven discrete types of risk groups (6 953 577 nodes). Risk group nodes were classified into three main categories—static, growing, or decaying—based on pre-defined transmission dynamics in the simulation (Supplementary Table S3), resulting in 4 619 000 static, 1 694 039 growing, and 640 538 decaying samples. Tree objects were imported into each model as delimited files containing branch-specific row information, which can be downloaded from the Github repository.

### 2.3 Sampling bias and model performance

Though the metrics used to describe tree shape (e.g. diversity) are potentially useful in classifying dynamic transmission clusters, each also has the potential to suffer from the reliance on the informativeness of the data, as with other phylogenetic and epidemiological approaches (Rife *et al.* 2017). Three attributes describing data informativeness have been incorporated into the models described below in order to evaluate the sensitivity of the models, as well as to allow the user to provide a measure of confidence in the results, given *a priori* knowledge of the data at hand. Included in this assessment were (1) sample size (number of individuals represented in the phylogeny) and (2) sampling frequency (percentage of original group size represented in the phylogeny). Additional information regarding the potential influence of these attributes on model performance is described in Supplementary Section SE.

### 2.4 Application to an HIV-1 subtype B outbreak

A phylogenetic tree representing 27 115 viral polymerase nucleotide sequences sampled from human immunodeficiency virus (HIV)-infected individuals in the state of Florida was reconstructed previously, as described in Rich *et al.* (2022). The phylogeny was also previously annotated according to transmission clusters identified using MicrobeTrace (Campbell *et al.* 2021). Clusters comprising purely monophyletic clades were used herein. That is, clades containing a mix of individuals identified as clustering according to MicrobeTrace and unclustered individuals were considered background, rather than clusters. Tree metrics not relying on molecular clock calibration were calculated for each cluster within the tree. The final tree, containing cluster identity information and tree metrics, was used as input for *DeepDynaTree*. Supplementary Figs S17–S19 describe the raw node features, normalized node features, and edge features, respectively. Missing node features were imputed with their mean value in the training set of ARI and TB combination data. The full phylogeny was also restricted to samples taken from 2012 to 2015 by trimming external nodes sampled between 2016 and 2017. Remaining internal nodes with only one child as a result of this trimming were also removed in order to maintain a bifurcating tree topology. Any clusters for which only one individual remained were also excluded from the analysis. It is important to note that no inference was made using the phylogenetic tree and patient metadata regarding directionality of transmission among individuals.

### 2.5 Input data pre-processing

Because the input distribution was highly unbalanced among the three classification categories, these data represent a realistic scenario in which sampling is biased according to prevalence within the population. Regardless, the data were re-distributed for model development by using a tree-based random split method with a ratio 60%–20%–20%. This splitting strategy was performed on ARI and TB simulations individually to keep the portion of ARI/TB trees the same for training/validation/test sets. Supplementary Section SC illustrates the detailed distribution of node, tree, and edge features used in different subsets for the model development and the success of providing a consistent data distribution over different subsets. It is important to note that in our experiments, 5-fold cross-validation was used for all machine learning based methods to select the optimal parameters. Considering the high computational cost introduced by deep learning, we have optimized all deep learning based methods on the above fixed validation data set, instead of using the 5-fold cross-validation. Before feeding the data into the learning algorithms, we performed standard data pre-processing for raw inputs, including quantile-based data discretization, one-hot encoding, and z-score normalization, based on different feature formats (see Supplementary Section SB for details).

### 2.6 PDGLSTM for improved dynamic transmission cluster prediction

Inspired by (Xu *et al.* 2017, Monti *et al.* 2018), we proposed a Primal-Dual Graph Long Short-Term Memory (*PDGLSTM*) model, as shown in Fig. 1b. The original phylogenetic tree is referred as the *primal graph*. Its dual graph (also known as line graph in graph theory) was constructed by considering each primal edge as a dual vertex, and two dual vertices considered adjacent if they shared a common endpoint in the primary graph. Supplementary Fig. S24 provides an illustration of the dual graph construction given an example phylogenetic tree. Following this process, *PDGLSTM* generates two disjoint sub-graphs for a single input tree, including a node-centric primal graph and edge-centric dual graph. Two modules were operated alternatively in order to learn the representations for both nodes and edges (1) primal-dual state updating and (2) primal-dual message passing. In state updating, two parallel LSTM modules were employed, respectively, on the primal and dual graphs, taking the pre-processed nodes and edge features as the initial input. As shown in Fig. 1b, Node-LSTM and Edge-LSTM take the pre-processed nodes and edge features as the initial input. Subsequently, the message passing operation between primal and dual graphs is performed to exchange the information of nodes and edges, wherein the output of a node’s LSTM is passed to its linked edges, and the node itself correspondingly receives the linked edges’ LSTM output as well. By repeating the message passing and updating steps iteratively, every node and edge can gather the updated states from multi-hop neighborhoods, resulting in enhanced and more informative hidden representation. In the last iteration step, only node states are updated by Node-LSTM, and the state of each node is used to predict the node type through a fully connected layer followed by a softmax activation, which generates the normalized probability for each cluster type. In following, we represent a phylogenetic tree using a tree structured graph notation G=(V,E), where $V=\left\{v_{1},\ldots ,v_{N}\right\}$ is a set of N nodes, and $e_{ij}\in E$ is an edge from node $v_{i}$ to node $v_{j}$. $v_{i}$ and $e_{ij}$ denote the features of node $v_{i}$ and edge $e_{ij}$.

#### 2.6.1 Primal-dual state updating

In the primary graph, each node LSTM takes its aggregated messages as current input and generates the updated node state by jointly considering the current the historic information. Formally, given the hidden state $v_{i}^{l}$ of node $v_{i}$ at time step l and its aggregated message $m_{i}$, the node state is updated following the $\mathrm{LST}M_{\mathrm{node}}$ updating rule:

(1) $$\begin{array}{l} f_{i}^{l}=\sigma (W_{f}^{n}m_{i}^{l}+U_{f}^{n}v_{i}^{l}+b_{f}^{n})\\ i_{i}^{l}=\sigma (W_{i}^{n}m_{i}^{l}+U_{i}^{n}v_{i}^{l}+b_{i}^{n})\\ o_{i}^{l}=\sigma (W_{o}^{n}m_{i}^{l}+U_{o}^{n}v_{i}^{l}+b_{o}^{n})\\ {\tilde{c}}_{i}^{l}=\sigma (W_{c}^{n}m_{i}^{l}+U_{c}^{n}v_{i}^{l}+b_{c}^{n})\\ c_{i}^{l+1}=f_{i}^{l}{}^{\circ}c_{i}^{l}+i_{i}^{l}{}^{\circ}{\tilde{c}}_{i}^{l}\\ v_{i}^{l+1}=o_{i}^{l}{}^{\circ}\sigma (c_{i}^{l+1}) \end{array},$$

where $W^{n},U^{n},b_{x}^{n}$ are model parameters for x∈{f,i,o,c}. $f_{i}^{l},i_{i}^{l},o_{i}^{l},c_{i}^{l}$ denote the output of forget gate, input gate, output gate, and memory cell, respectively. Here, the time step l represents the l-th primary-dual graph communication and it is conceptually similar to the layer index l used by other GNN variants.

Similarly, in the edge-centric dual graph, the edge state $e_{ij}^{l}$ at time step l is also updated using a LSTM model:

(2) $$e_{ij}^{l+1}=\mathrm{LST}M_{\mathrm{edge}}(m_{ij}^{l},e_{ij}^{l}),$$

where the $m_{ij}$ represents the aggregated message for edge $e_{ij}$ and the detailed computation of LSTMis as same as Equation (1), except replacing the node inputs with the edge and its associated message information. In our setting, the hidden sizes of both Node- and Edge-LSTM models are configured as 64.

#### 2.6.2 Primal-dual message passing

Primal-dual message passing strategy is designed to aggregate and process the corresponding messages for nodes and edges, allowing for information fusing between the node set V and the edge set E. Specifically, for node $v_{i}$, its aggregated message $m_{i}$ is computed from the current states of inbound edges and node itself:

(3) $$m_{i}^{l}=\mathrm{LeakyReLU}\left(W_{N}\left[v_{i}^{l},\sum_{j:j\to i}e_{ji}^{l}\right]+b_{N}\right),$$

where, j→i denotes all the inbound edges to the node $v_{i}$.

Similarly, the message information $m_{ij}$ for edge $e_{ij}$ is generated by:

(4) $$m_{ij}^{l}=\mathrm{LeakyReLU}(W_{E}[v_{i}^{l},e_{ij}^{l},v_{j}^{l}]+b_{E}),$$

where both W and b are learnable parameters.

#### 2.6.3 Transmission cluster prediction

With iteratively applying the primal-dual message passing and state updating steps, *PDGLSTM* is able to gather extensive information from multi-hop neighboring nodes and edges, resulting in an enhanced and more informative node representation for final prediction. In our setting, the above two steps were repeated 13 times, which means the receptive field of each node was up to 13-hop neighboring nodes and 13 edges. A prediction head with one fully connection layer was utilized on the final high-level node embedding.

(5) $$p(v_{i})=\mathrm{Softmax}(f(v_{i}^{L})),$$

where f is realized by a single-layer feed-forward network, and L is the total time steps. The final status of clusters is then generated by averaging the predicted probabilities of each individual node.

### 2.7 Model evaluation metrics

*DeepDynaTree* was comprehensively evaluated on seven performance metrics including macro-averaged precision, F1-score, and area under the receiver operator characteristic curve (AUROC), balanced accuracy, Matthews Correlation Coefficient (MCC), Brier score (BS), and cross entropy (CE). Considering the extremely unbalanced label distribution in our data set, we utilized macro-averaging strategies, which first generated the metric values for each class in the one-vs-rest manner, and then averaged them with equal weights to give the same importance to each class.

### 2.8 Baseline models and implementation details

We compared 7 node-based methods and 3*DeepDynaTree* baseline approaches with our final *DeepDynaTree-PDGLSTM* model. The node-based methods only utilized the tree shape metrics as input for prediction, including logistic regression (LR) (Hosmer *et al.* 2013), random forest (RF) (Breiman 2001), Extreme Gradient Boosting (XGboost) (Chen and Guestrin 2016), multilayer perceptron (MLP) (Haykin 1994), DeepSet (Zaheer *et al.* 2017), SetTransformer (Lee *et al.* 2019), and TabNet (Arık and Pfister 2020). The *DeepDynaTree* baseline approaches include Graph Convolutional Network (GCN) (Kipf and Welling 2017), Graph Attention Network (GAT) (Veličković *et al.* 2018), and Graph Isomorphism Network (GIN) (Xu *et al.* 2019). A detailed introduction of the baseline methods is incorporated in Supplementary Section SF. *DeepDynaTree-PDGLSTM* model took approximately 3 days to train for around 300 epochs with mini-batch of size 32. An adam optimizer was used for training with an initial learning rate $3\times 10^{-3}$, and 90% reduction was applied to the learning rate if the validation loss did not improve in the consecutive 50 epochs until reaching the minimum value $1\times 10^{-6}$. We fine-tuned all the hyper-parameters on the validation data set. All experiments were performed on a workstation with 12 Intel Core i7-5930K CPUs and a single Nvidia GeForce GTX TITAN X GPU card.

## 3 Results

### 3.1 Method performance

**Figure 2.**
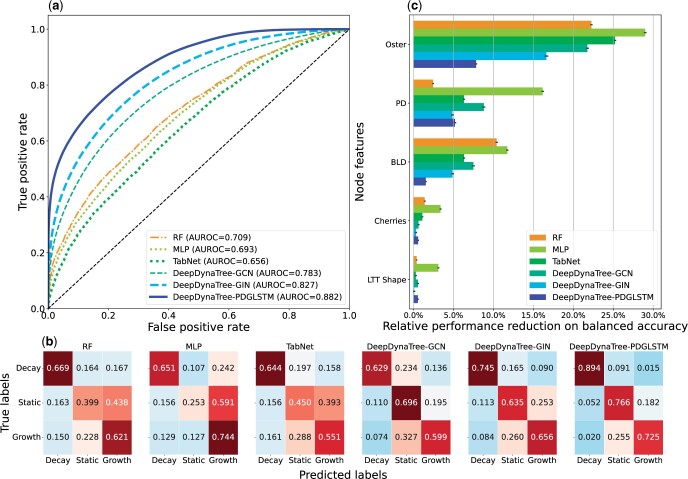
Figurative performance comparison of six selected competitive classifiers and permutation feature importance results. (a) Comparison of various classifiers, shown on macro averaged receiver operator characteristic curves (ROC), with corresponding AUC. (b) Comparison of various classifiers, shown on confusion matrices, and elements were row-wise normalized by class support size. (c) Relative permutation feature importance results measured by the balanced accuracy. *x*-axis represents the relative performance reduction in percentage compared to the non-permutated model and *y*-axis represents the top-5 most important tree shape metric features.

This section compared various prediction methods, as well as a quantitative evaluation of the importance of different tree shape metrics in dynamic transmission cluster classification tasks. Figure 2a and b and Table 1 demonstrated the comparison of ROC curves, confusion matrices, and seven metrics for the global classification performance on the simulated test set, including outbreaks of acute and chronic respiratory infections. *DeepDynaTree* models outperformed all node-based methods over all the evaluation metrics by a large margin. Random Forest (RF) could be considered a reference point of node-based methods with the best performance. When compared to RF, even the baseline GAT model within *DeepDynaTree* achieved 5.8% and 2.8% relative improvement compared with RF in accuracy and AUROC, respectively. With *PDGLSTM*, the improvements further extended to 39.7% and 24.4% for the above two metrics. A similar trend was also observed when considering the remaining metrics, as detailed in Table 1. These results suggested that *DeepDynaTree* shows a distinct improvement in transmission cluster prediction capacity over the three target cluster types (growing, static, decaying).

**Table 1.** Performance for classification of dynamic transmission clusters in the test dataset compared with seven node-based methods, four GNN variants, and two ablation models.^a^

Methods	Models	Balanced accuracy ↑	Macro F1 ↑	Macro Precision ↑	Macro AUROC ↑	MCC ↑	BS ↓	CE ↓
Node-based models	LR	0.525	0.429	0.437	0.675	0.173	0.614	1.016
	RF	0.569	0.469	0.479	0.709	0.235	0.581	0.949
	XGBoost	0.548	0.454	0.472	0.699	0.214	0.618	1.023
	DeepSet	0.484	0.390	0.408	0.635	0.127	0.626	1.012
	SetTransformer	0.486	0.388	0.410	0.637	0.132	0.632	1.020
	MLP	0.549	0.411	0.472	0.693	0.204	0.650	1.029
	TabNet	0.549	0.461	0.464	0.656	0.208	0.584	0.952
DeepDynaTree	GAT	0.602	0.515	0.518	0.729	0.277	0.551	0.891
	GCN	0.641	0.594	0.572	0.783	0.390	0.472	0.795
	GIN	0.679	0.604	0.579	0.827	0.404	0.459	0.755
	PDGLSTM	0.795	0.746	0.715	0.882	0.569	0.327	0.525
	PDGLSTM w/o [D]^b^	0.675	0.643	0.627	0.822	0.477	0.438	0.753
	PDGLSTM w/o [L]^c^	0.730	0.624	0.598	0.753	0.444	0.453	0.711

a The selected node-based models included Logistic Regression (LR), Random Forest (RF), Extreme Gradient Boosting (XGBoost), DeepSet, SetTransformer, Multilayer perceptron (MLP), and TabNet. Under our proposed *DeepDynaTree* framework, the selected GNN variants included Graph Attention Network (GAT), Graph Convolutional Network (GCN), Graph Isomorphism Network (GIN), and the newly designed Primal-Dual Graph Long Short-Term Memory (*PDGLSTM*). The best performance of each evaluation metric within two different methods is represented in bold.

b PDGLSTM without the dual graph design.

c PDGLSTM without LSTM module to maintain the historic state information.

Compared with all the current GNN variants, *PDGLSTM* achieved the best performance on all evaluation metrics (Fig. 2a and Table 1). Taking the second-best model (GIN) as a strong competitor, *PDGLSTM* enhanced the accuracy and AUROC from 67.9% to 79.5% and 0.827 to 0.882, respectively. *PDGLSTM* also remarkably outperformed GAT by relative improvements of 32.1% and 21.0%. Additionally, the confusion matrices shown in Fig. 2b further demonstrated that the *PDGLSTM* outperformed all GNN competitors on all cluster types. For example, 76.6% of static, 89.4% of decaying, and 72.5% of growing clusters were correctly predicted by *PDGLSTM*, whereas the GIN model correctly identified 63.5%, 74.5%, and 65.6% of clusters, respectively, and the performance of GCN further dropped to 69.6%, 62.9% and 59.9%, respectively. These results demonstrated that explicitly framing the time and genetic distance information into the edge-centric dual graph learning process, rather than only utilizing the connectivity of transmission clusters, effectively improved the representation learning capacity of GNNs, leading to more accurate inferences. The figurative performance comparison of all eleven models is illustrated in Supplementary Fig. S20.

#### 3.1.1 Feature importance

Feature (in this case, tree shape metric) importance was defined to be the decrements of model performance when a single feature value was randomly shuffled (50 times in this study). Hence, the larger the decremental decrease, the more significant the feature was considered to be to model performance. As demonstrated in both Fig. 2c and Supplementary S21 and S22, the metric (“Oster”) originally used to describe human immunodeficiency virus (HIV) transmission rates (Oster *et al.* 2018, Rich *et al.* 2020) and phylogenetic diversity (“PD”) were ranked highest for *PDGLSTM*, as well as the other GNN and node-based models. These values were also highly correlated (ranked correlation coefficients >0.65 in Supplementary Fig. S4). The relative feature importance of the Oster statistic (7.84% reduced balance accuracy with permutation) over PD was 5.19%, indicating this statistic represents the most reliable of the tree metrics and is unrivaled in distinguishing transmission cluster dynamics among the metrics chosen in this study. Branch length differences (BLD) over time, the number of cherries, and the characteristic shape of lineages through time (LTT) outperformed the remaining previously published metrics; however, they contributed primarily to node-based models, with minimal contribution to *DeepDynaTree*-specific models.

#### 3.1.2 Model ablation study

Two important designs of our *PDGLSTM* model are (1) iterative primary-dual graph communication and (2) node and edge state updating with temporal information. To demonstrate their effectiveness, we performed associated ablation studies. For the first design assessment, we removed the edge-centric dual graph [D] so that the model was restricted to the basic connectivity information and passed the message between nodes only. As shown in the lower section of Table 1, BS increased by 33.9% and balanced accuracy dropped by over 15% with this removal. To evaluate the second design, we replaced the parallel LSTM models [L] with the parallel fully connected layers so that the states of the node or edge at the next step were only determined by the current states and received messages without any historical information. BS with this replacement increased by 38.5%, and balanced accuracy was reduced by 8.2% from the original model. The reduced performances confirm the effectiveness of both model designs and suggest their equal importance.

#### 3.1.3 Model limitation analysis

To pinpoint the limitations of the *PDGLSTM* model for future application, we calculated the predicting accuracy distribution over ground truth cluster characteristics, including sampling fraction, size, and time span of the cluster (Supplementary Fig. S23a). The non-linear shape of the weighted cross entropy loss (WCEL) across sampling fraction intervals was consistent with the relationship described in Supplementary Section SA—i.e. sampling thresholds exist, below and above which classification is unreliable. Several features included in the model—$R_{0}$, fraction of time spent in growth phase, and $R_{\max}$—are derived from coalescent estimates of $N_{e}$. Owing to the assumption of the coalescent model that the sample size is much smaller than the true population size (Kingman 1982a,b), over-sampling can bias $N_{e}$ estimates and, consequently, the above-described features. On the other end of the spectrum, it is apparent that under-sampling can provide too few data points for reliable feature estimation. Increased (unfavorable) WCEL was similarly observed for small and large values of cluster size, but only small values corresponding to shorter time spans were considered to result in WCEL (Supplementary Fig. S23). Based on the confidence values of WCEL, reliable results could be achieved with clusters as small as 47 sampled individuals and sampling as sparse as 22% of the risk group population when the sampling time span was over the course of only one month. It is important to keep in mind, however, that reliability declines when the cluster size is <47 (or >502) individuals and/or the cluster has occurred over a time span of <34 days (from first sampled infected individual to most recent). Time span was highly correlated with the Oster statistic (coefficients = 1), indicating a link between transmission rates and the maintenance of infection among a risk group within a population over time. Exhibiting a similar pattern in WCEL, Oster values <34 were considered to provide unreliable classifications using the model. Owing to its relationship with time span, however, the calculation of Oster values for clusters prior to assessing confidence in *DeepDynaTree* classification can be accurately replaced with knowledge of the cluster time span. PD values implicated a threshold of 515 for a reliable prediction and can similarly be used to determine *a priori* the reliability of the model classification.

The parameters used to describe transmission characteristics for each risk group in the simulation were similarly evaluated for their impact on prediction accuracy, demonstrating again the slight difficulties in discriminating certain static, growing, and decaying clusters for the *PDGLSTM* model (Supplementary Fig. S23c), though improved dramatically over remaining models. When evaluated in more depth, higher initial contact numbers [$N_{0}$ in Supplementary Section SD, Equation (1)] among individuals within a cluster posed a challenge for discrimination, as did intermediate probabilities of transmission and initial estimates of $R_{0}$ (see Supplementary Table S3). However, this finding was not entirely surprising, as static and growing clusters were allowed to form with similar transmission characteristics, only to differentiate later in time, during which tree topology data are important.

### 3.2 Model application to Florida HIV epidemic

As part of extensive surveillance efforts in the state of Florida, a total of 28 098 partial HIV-1 pol sequences were collected during 2012 to 2017 and were considered for transmission cluster analysis in Rich *et al.* (2022). Of these, 27 115 (96.5%) were classified as subtype B and included in subsequent analyses. Among these sequences, 4943 (18.2%) clustered with at least one other, 20.8% of which were considered to comprise large clusters (11–70 individuals) and were prioritized for assessment of putative risk factors using patient metadata provided from the Florida Department of Health (FDOH). These data included year of HIV genotype determination, year of birth, birth region, gender at birth, race/ethnicity, county of residence, and mode of transmission exposure. In this previous study, the identified larger clusters tended to originate from persons living with HIV (PWH) diagnosed in more recent years. Cluster size was also associated with the age of PWH, their gender, and mode of transmission. We therefore sought to use these data as an opportunity to determine the relationship of transmission dynamic prediction with cluster size and demographics.

**Figure 3.**
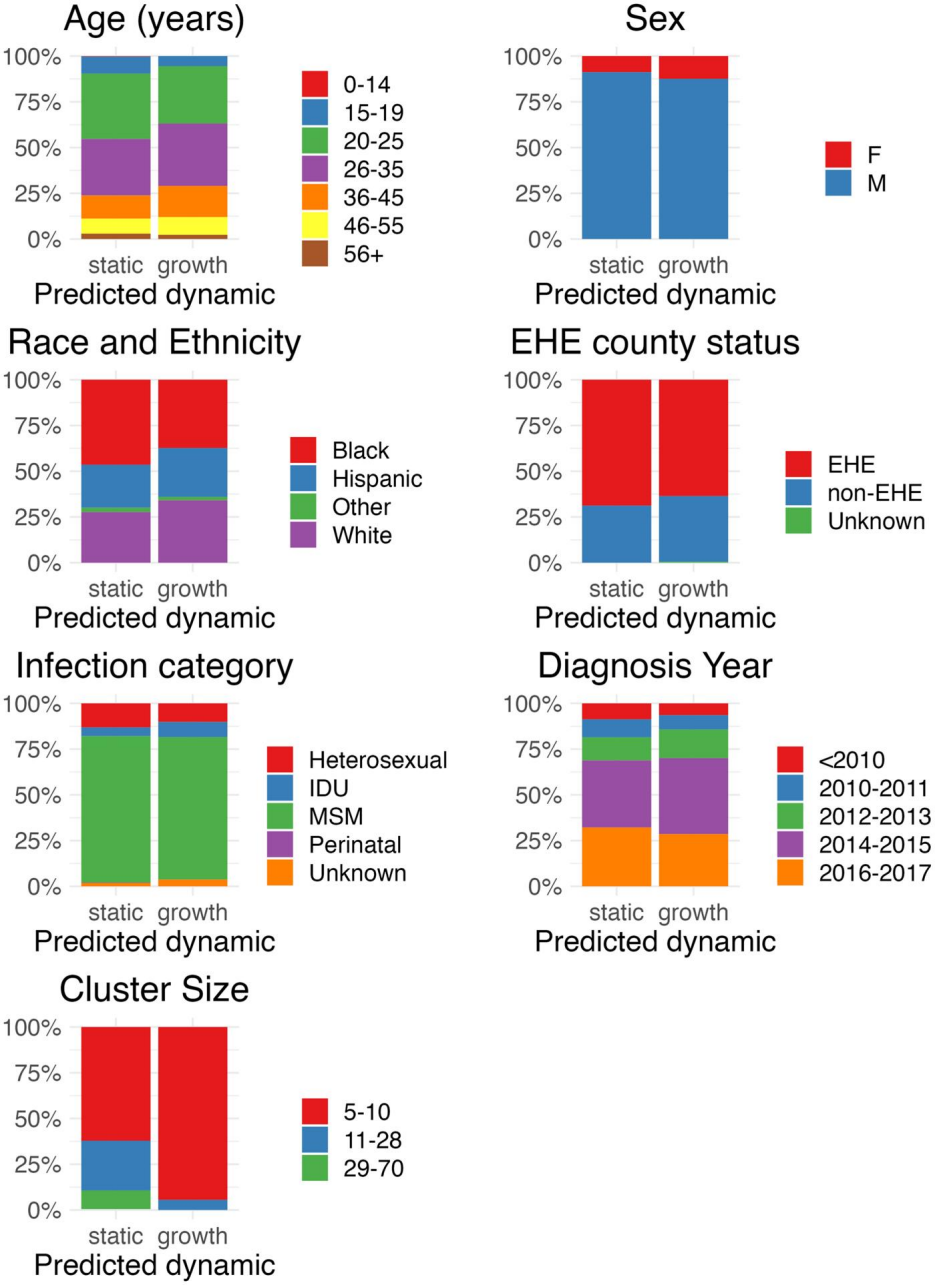
Demographic and cluster size distributions among HIV transmission clusters classified according to transmission dynamic using *DeepDynaTree-PDGLSTM*. A total of 4943 HIV-infected individuals from the state of Florida were considered to cluster with at least one other individual according to MicrobeTrace (Campbell *et al*. 2021) analysis by Rich *et al*. (2022). Transmission dynamic (decay, static, growth) was assigned to each cluster in this study using *DeepDynaTree*. Surveillance data were provided for each individual within a given cluster, for which the relationship to transmission dynamic is shown here. Original cluster sizes are also shown. EHE, End-the-Epidemic program priority assignment of Florida counties; MSM, men who have sex with men; IDU, intravenous drug use; Other race, American Indian/Alaska Native, Asian, Native Hawaiian/Pacific Islander, or Multi-race.

Following model application using *DeepDynaTree-PDGLSTM*, clustered individuals within this outbreak were represented by static (83%) and growing (17%) transmission only (no decaying clusters were observed, Fig. 3). Contrary to what might be expected, larger cluster sizes (11–70 individuals) were associated with static transmission, whereas smaller clusters (≤10 individuals) tended to be classified as growing (Fig. 3). Additionally, the estimated number of infections per 100 person-years (Oster) for these smaller, yet growing clusters tended to be larger (>46) than for clusters classified as static (Fig. 3).

In order to assess the robustness of *DeepDynaTree* and its utility in the earlier stages of an outbreak, the above phylogeny was restricted to samples taken only from 2012 to 2015. Consistent with the above observations using *DeepDynaTree-PDGLSTM* on the entire HIV dataset, clustered individuals were categorized as 84% static, 15.9% growing, and 0.1% decaying transmissions, resulting in identical prediction for 95.2% of individuals as compared with predictions using the full phylogeny. Discrepancies were explained by a transition of 3.7% of nodes from static to growth, suggesting a <5% false positive rate for estimation of the fraction of the population contributing to increased spread of HIV. This value may not fully represent a false positive rate, however, as overall static clusters may have experienced brief, early periods of growth masked by static transmission if sampled over a relatively long period of time. It is also important to remember that the model of growth used in training *DeepDynaTree* was parametric (linear) and so would not be able to classify more complex dynamics.

## 4 Discussion and conclusion

Identification of transmission dynamics among risk groups of pathogen infection is of utmost importance to public health surveillance, providing retrospective understanding of the driving factors of spread and real-time insight into the impact of public health interventions. Whether it be modifications in risk group behavior, the pathogen genome itself, or to the availability of resources, the impacted transmission trajectory can be altered, even within a subset of infected individuals. When traced in the context of patient and environmental metadata (e.g. geographical origins and vaccine availability), these alterations provide critical information that aids in how to combat further spread or future pandemics caused by similar pathogens. Whereas sampled pathogen sequence data, specifically the phylogenetic relationships among these sequences, have been utilized in the estimation of relevant population genetic and/or epidemiological parameters (e.g. $R_{0}$), we demonstrate that these estimates are unreliable for smaller datasets, such as individual transmission clusters, even when combined into complex models (e.g. Random Forest). We proposed instead to learn these dynamics jointly from commonly used tree metrics and the underlying tree data by developing a phylogenetic-informed neural network platform—*DeepDynaTree*. We further propose a novel variant of the long short-term memory neural network approach (*PDGLSTM*), which performs iterative message passing between the primal and dual graphs along the phylogenetic tree’s topological structure, utilizing information from both connectivity of the nodes and branch lengths. When integrating these multiple sources of information, the *PDGLSTM* model is capable of generating more informative representation of transmission clusters and enhanced predictions of cluster behavior in terms of growth or decay (or lack thereof).

When applied to empirical data derived from an HIV subtype B outbreak in the state of Florida, *DeepDynaTree* determined that previously identified transmission clusters were represented by majority static transmission, followed by a minority of clusters characterized by transmission growth. The absence of decaying clusters in this outbreak suggests current public health interventions may not be acting to reduce transmission among sampled individuals. Importantly, the inverse relationship between cluster size and predicted transmission growth suggests cluster size may not be a reliable indicator of spread within a risk group. A focus on larger clusters, assuming they represent a greater risk of transmission, could inform a different, and potentially ineffective, strategy for future sampling and targeted public health interventions.

As with every analytical approach, *DeepDynaTree* is not without its limitations; we have carefully evaluated the performance of the *PDGLSTM* model so as to aid users in understanding the boundaries of reliability during application. For example, users should consider additional surveillance information when interpreting results for smaller transmission clusters (comprised of <47 individuals) and/or encompassing a brief sampling time span of <34 days. Whereas the model was not particularly sensitive to the fraction of the cluster being sampled for sequence analysis, sampling was performed randomly rather than using a time- or size-dependent strategy, which may be more realistic of surveillance data and should be taken into account during analysis as well as further training of *DeepDynaTree*. Growing and decaying clusters in these simulations were also modeled using a highly deterministic model of changing population size, whereas the true time-varying transmission dynamics for risk groups may differ depending on stochastic outside factors that influence individual or virus behavior. A broader simulation study encompassing a wider range of time-varying growth models would not only capture a more realistic epidemic, but allow for a more fine-grained transmission prediction, including growth types and rates. It is also important to note that the tree topologies simulated for training and validation were known, whereas phylogenies reconstructed from real-world genetic sequence data are often accompanied by a degree of uncertainty and error. Additional investigation into the influence of this uncertainty on model inferences is warranted. Despite these limitations, we describe the first, and thus most extensive, dynamic cluster simulation set thus far, for which *DeepDynaTree* was able to classify transmission dynamics with >79% accuracy, surpassing the <40% accuracy achieved with non-neural network models. This accuracy was achieved, despite inclusion of low-performance features, such as the lineage-through-time shape. Whereas model retraining following feature selection would reduce the unnecessary dimensionality of *DeepDynaTree*, it is important to keep in mind that the prioritization of this study on the early, exponential phase of an epidemic may not reflect feature importance when applied retroactively to more endemic pathogens. Additional investigation into the relevance of these features for risk group dynamics within longer-spanning outbreaks is warranted.

*DeepDynaTree* classifies specific groups of individuals as growing, decaying, or static, taking as input *user-specified* transmission clusters, which can be identified using any number of phylogenetic-based cluster-identification algorithms (e.g. Prosperi *et al.* 2011, Ragonnet-Cronin *et al.* 2013). By doing so, the assumption is made that input clusters have been identified correctly; however, the accuracy of these tools in identifying dynamic clusters has yet to be determined. These approaches are also *themselves* limited in their reliance on thresholds for summary statistics of pairwise genetic distances among samples, which have been demonstrated to differ substantially across transmission pairs and clusters in the study of human immunodeficiency virus (HIV) (Ratmann *et al.* 2016, Chato *et al.* 2020). These differences are influenced by factors such as the extent of clinical follow-up (Chato *et al.* 2020) and fraction of the risk group sampled (Novitsky *et al.* 2014, Poon 2016, Dasgupta *et al.* 2019) and provide the potential for the propagation of error in cluster analysis. Additional training of *DeepDynaTree* for not only characterization, but identification, of dynamic transmission clusters among sampled individuals within a phylogeny may improve our ability to detect clusters of interest and should be explored. Founded on well-developed deep learning techniques, *DeepDynaTree* allows for continual learning of both ground truth and empirical data as they become increasingly available with elevated pathogen sequencing efforts.

## Supplementary Material

vbae158_Supplementary_Data

## Data Availability

The code used to generate the simulation data is available in https://github.com/salemilab/DeepDynaTree. We received HIV sequence data and metadata extracts from FDOH in a fully de-identified format compliant to the Health Insurance Portability and Accountability Act (HIPAA). For replication purposes, data request to the FDOH (Research@flhealth.gov) can be made according to state, federal regulations and compliance with required ethical and privacy policies, including IRB approval by FDOH and execution of data user agreements. Requests are independently reviewed by FDOH.
