# Supplementary material for: Phylogenetic-informed graph deep learning to classify dynamic transmission clusters in infectious disease epidemics
Source: Bioinform Adv. 2024 Nov 7;4(1):vbae158. doi: 10.1093/bioadv/vbae158 (PMC11552518; doi:10.1093/bioadv/vbae158)
Supplement: vbae158_Supplementary_Data [file vbae158_supplementary_data.pdf]

Supplementary Material

A. Generalized tree shape metrics evaluated in prediction of cluster dynamics

The information contained in the shape of a molecular phylogeny can be crudely partitioned into two components - the topology (branching structure) and branch lengths (also sometimes referred to as node heights). Although these components are intimately linked, the distinction is nevertheless useful (Mooers and Heard, 1997). Some tests of evolutionary hypotheses require only tree topology, such as meterics that measure tree balance (Frost and Volz, 2013), whereas other tests, particularly those based on the birth-death or coalescent processes (Nee et al., 1994, 1995), depend only on branch length information. The common feature of these tests is that they can be used to estimate the rate (or change of rate through time) of evolutionary processes. For example, when assuming a clock-like evolutionary behavior (i.e., the rate of accumulation of mutations remains constant over time), branch lengths representing genetic differences between samples taken at a specific point in time can be re-scaled in similar units of time, revealing divergence dates, and thus divergence rates over time, among lineages within the tree. Nee *et al.* (Nee et al., 1995) proposed the lineage through time (LTT) plot as a way to graphically investigate the demographic history of a population using a sample of gene sequences and to estimate birth and death rates in order to test the null hypothesis of a static population. Pybus and Harvey soon after observed that this constant-rates model could be rejected for a given phylogeny if the internal nodes, or divergence events, were closer to the root of the phylogeny than would be expected under a pure birth model (virtually no extinction of lineages) (Pybus and Harvey, 2000). This observation was used to develop a statistic (γ) that described the relative position of the nodes within the tree and was used in this study to quantify transmission dynamics. However, it is important to note that the gamma statistic assumes equidistant root-to-tip branch lengths (i.e., all samples were taken at the same time), which is violated in our simulations and in modern epidemic datasets. An additional statistic unbiased by sampling time was thus included ("BLD"), which was calculated as a function of the difference between branch lengths (median) observed in the first and second halves of the time at which the cluster was observed.

Pybus later improved on the LTT plot to provide a framework for inference of the population demographic history (Pybus et al., 2000), which has since undergone several changes, including the implementation of a Bayesian approach employing prior distributions for parameters pertaining to the underlying evolutionary process, as well as the change in effective population size (N_e) (Drummond et al., 2005) in order to account for uncertainty in tree reconstruction. The original "skyline" family of Bayesian phylogenetic frameworks have assumed that N_e follows a stochastic process such as a Brownian motion (Pybus et al., 2000; Minin et al., 2008), which was demonstrated by Volz and Didelot (Volz and Didelot, 2018) to have a potentially large impact on estimates, especially when genealogical data are sparse and uninformative. Volz and Didelot (Volz and Didelot, 2018) thus recently proposed a modified approach defined according to a growth rate prior that was able to reproduce dynamics estimates (e.g., the initial rate of secondary infection [R_0]) expected under a tested variety of epidemic situations, including otherwise erroneously predicted stable populations. For the simulations described herein, Volz and Didelot's approach, referred to as *skygrowth*, was used to estimate the effective population size (N_e) for individual risk groups within the tree in order to derive point estimates of potentially relevant epidemic parameters, including R_0, rate of overall N_e growth ("AbsGrowthRate"), maximum rate of growth given discrete intervals of time ("R_{max}"), and fraction of time spent in the maximum growth phase ("FractionTimeGrowth") (Table 1).

Branch lengths scaled in time have also been used in other ways to characterize epidemic dynamics. Recently, Oster *et al.* demonstrated that human immunodeficiency virus (HIV) transmission rates for a given cluster (number of transmissions per 100 HIV-infected persons per year) could be estimated as a function of the size in tips, the sum of the branch lengths, and the length of the longest branch in the cluster (Oster et al., 2018; Rich et al., 2020). While only described in the context of a snapshot in time, we aimed to characterize the capability of the "Oster" statistic to distinguish the three classifications of transmission clusters described above (static, growing, and decaying). Testing the hypothesis that this statistic is primarily driven by the sum of the branch lengths, or phylogenetic diversity (PD), this value was also included in the model.

In terms of tree topology, the degree of balance, or symmetry, within the tree has been linked to phylodynamic inference, as it is thought to be influenced by biological factors such as differences in infectiousness or contact rate (Dearlove and Frost, 2015). One measure of imbalance relies on the simple identification of a branching pattern referred to as a cherry. Cherries are defined as two tips that share, or coalesce via, a *direct* ancestor within the tree. The expected number of cherries in a tree with n taxa under a pure birth (Yule) model is $n/3$ (McKenzie and Steel, 2000). In an asymmetric tree, tips tend to coalesce with branches deeper in the tree, and there are fewer cherries than expected. Using simulations of differing risk of transmission, Frost and Volz *et al.* (Frost and Volz, 2013) demonstrated that higher infectiousness resulted in more asymmetric trees (not observed here), though dependent to an extent on the sampling fraction (also not observed here). As dynamic risk groups were not investigated, we sought to explore whether the proportion of cherries (relative to the number of tips) for a given cluster could aid in the resolution of epidemic dynamics for a given cluster.

B. Feature description & pre-processing

Based on different raw data formats, we performed corresponding data pre-processing steps. Specifically, for the categorical feature *LTT Shape* with four categories in its raw format, i.e., concave, concave_convex, convex, and convex_concave, we utilized a one-hot vector for encoding it. For the continuous feature *gamma* but with infinity value (denoted by inf), we selected to use quantile discretization to split it into four groups, i.e., $(-71478.28, -4.184]$, $(-4.184, -1.154]$, $(-1.154, 0.916]$, and $(0.916, inf)$.Then one-hot encoding with four dimensions was utilized to represent different groups. The other continuous variables with limited data ranges including Oster, PD, AbsGrowthRate, FractionTimeGrowth, R_{max}, Cherries, BLD and R_0 were simply processed with a z-score normalization where each feature was normalized to the zero mean and unit variance. For the two edge features, we first applied inverse hyperbolic sine (short for ArcSinh) transformation, which can approximate the natural logarithm of the raw values and retain zero-valued observations, then standardize them with z-score normalization. Table 1 and 2 respectively provide the data statistics for raw node and edge features on combination of ARI and TB simulations.

Table 1. Summary statistics for generalized tree metric features.

Features	Descriptions	Min	Max	Mean	Std
Sampling Fraction	Fraction of total infected individuals sampled from a cluster	5.000×10^{-2}	1.000	2.067×10^{-1}	1.689×10^{-1}
Cluster Size	Total number of infected individuals in a cluster	5.000	3.988×10^{3}	1.458×10^{3}	5.073×10^{2}
Time Span	Time range from earliest sampled individual to most recently sampled individual within a cluster	3.000	2.370×10^{2}	1.168×10^{2}	5.694×10^{1}
γ	Pybus's γ (Pybus et al., 2000)	-3.028×10^{5}	inf	inf	NA
Oster	Estimated transmission rate (per 100 infected individuals per time unit) (Oster et al., 2018)[1]	3.106	2.370×10^{2}	1.168×10^{2}	5.694×10^{1}
PD	Phylogenetic diversity (sum of branch lengths scaled in genetic distance)	1.500×10^{1}	3.307×10^{4}	2.322×10^{4}	9.728×10^{3}
R_0	Basic reproductive number	3.359×10^{-5}	8.006	1.880	8.562×10^{-1}
Abs Growth Rate	Average growth rate for estimated N_e[2]	1.338×10^{-1}	1.437×10^{8}	3.812×10^{2}	1.469×10^{5}
Fraction Time Growth	Fraction of cluster time span (includes TMRCA[3]) spent in growth phase	0.000	1.000	4.891×10^{-1}	3.001×10^{-1}
R_{max}	Maximum rate of growth in estimated N_e[2]	-6.620	3.133×10^{6}	5.859×10^{3}	5.715×10^{3}
LTT Shape	Shape of LTT[4]	NA	NA	NA	NA
Cherries	Number of cherries per total number of taxa in cluster	7.692×10^{-2}	4.762×10^{-1}	3.037×10^{-1}	2.600×10^{-2}
BLD	Branch length difference	-3.100×10^{1}	1.500×10^{1}	1.291	1.360

[1] Originally proposed for HIV-infected individuals
[2] Effective population size
[3] Time of the most recent common ancestor
[4] Lineages through time (LTT) shape is classified as concave, convex, or a mixture of convex and concave, representing a non-numeric feature with no descriptive statistic information.

Table 2. Summary statistics for edge features.

Features	Descriptions	Min	Max	Mean	Std
Weight1	Time (days)	0.000	1.770×10^{2}	8.222	7.005
Weight2	Genetic distance (substitutions/site)	0.000	1.742×10^{-1}	7.400×10^{-3}	6.336×10^{-3}

C. Feature distribution

Figures 1 and 2 illustrate the distributions of the raw and normalized tree metric features on both training (including validation) and testing datasets for ARI and TB combinations. The three ground truth cluster characteristics, i.e. Sampling Fraction, Cluster Size and Time Span, are also included. Figures 4 and 5 respectively exhibit the Spearman's rank and Pearson correlation between tree shape metric features (including the three ground truth cluster characteristics). Figure 6 illustrates the distribution of the two edge features. Figure 3 exhibits the statistic information of the three dynamic transmission cluster types in training and test datasets. The above analysis for individual simulations are also provided, i.e., Figures 7, 8, 9, 13, 15 for ARI and Figures 10, 11, 12, 14, 16 for TB.

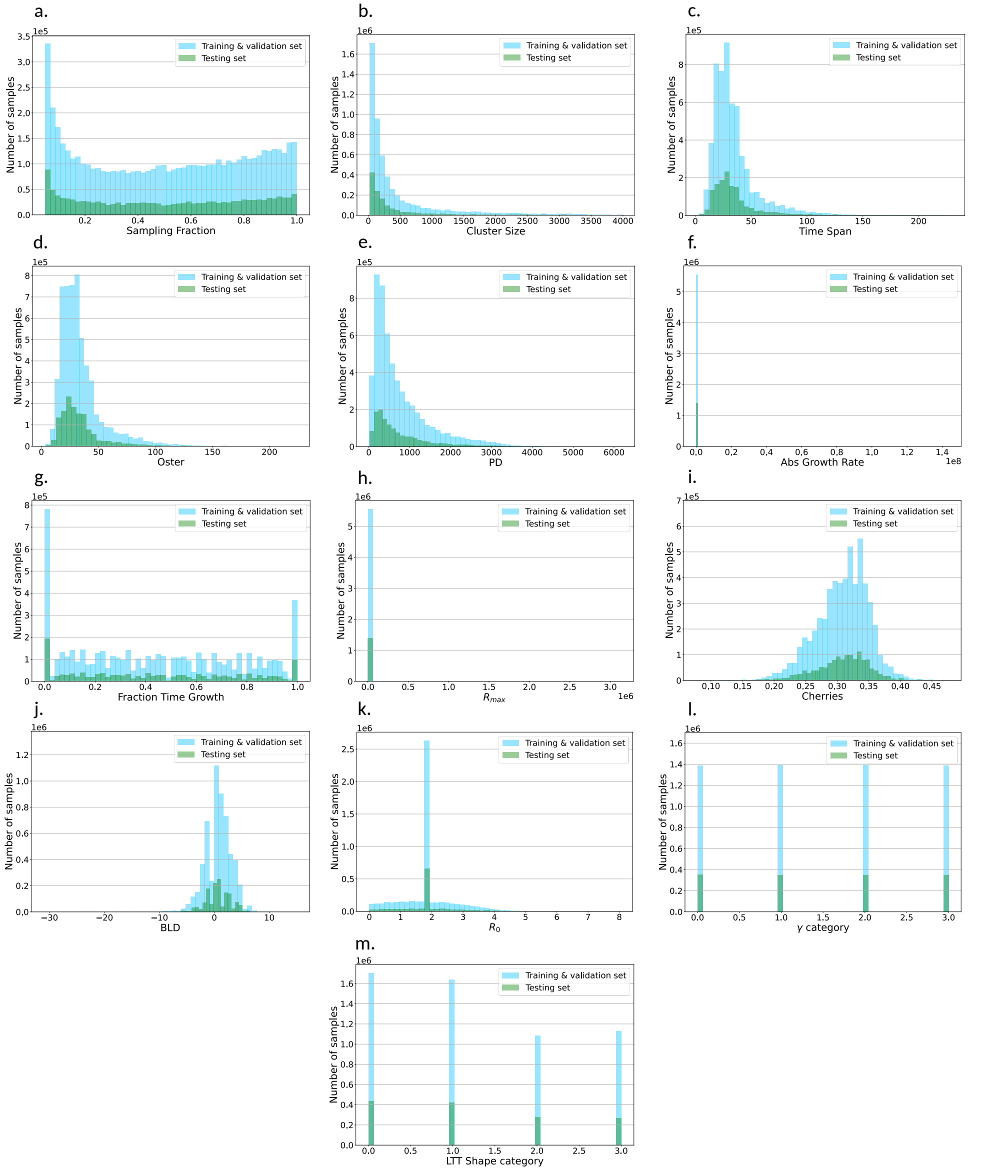

Fig. 1. Distribution of the raw tree shape metrics with numerical values on combination of ARI and TB simulations, including **a,** Sampling Fraction, **b,** Cluster Size, **c,** Time Span, **d,** Oster, **e,** PD, **f,** Absolute Growth Rate, **g,** Fraction Time Growth, **h,** R_{max}, **i,** Cherries, **j,** BLD, **k,** R_0, **l,** γ category and **m,** LTT shape category.

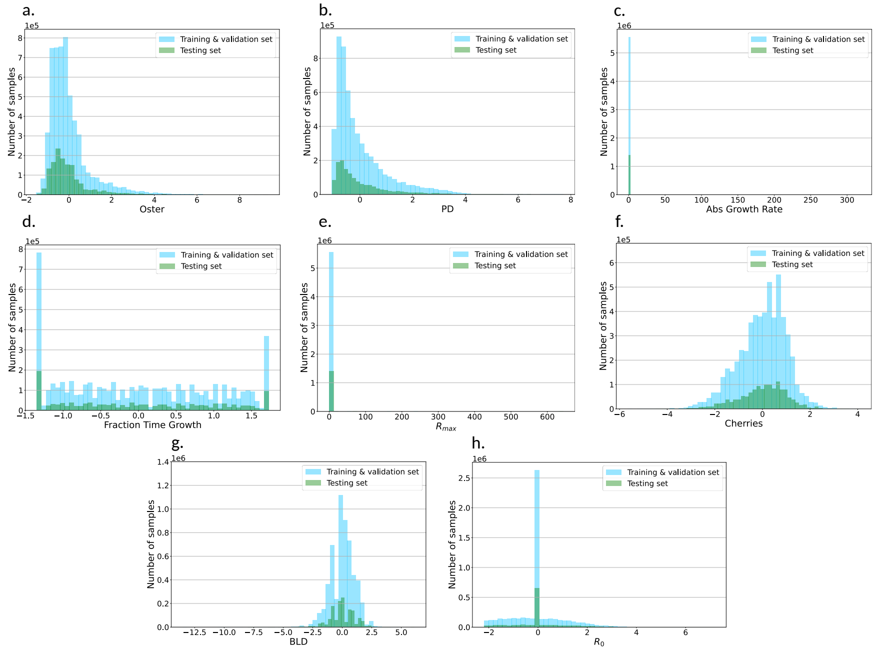

Fig. 2. Distribution of the normalized tree shape metrics with numerical values on combination of ARI and TB simulations, including **a,** Oster, **b,** PD, **c,** Absolute Growth Rate, **d,** Fraction Time Growth, **e,** R_{max}, **f,** Cherries, **g,** BLD, and **h,** R_0.

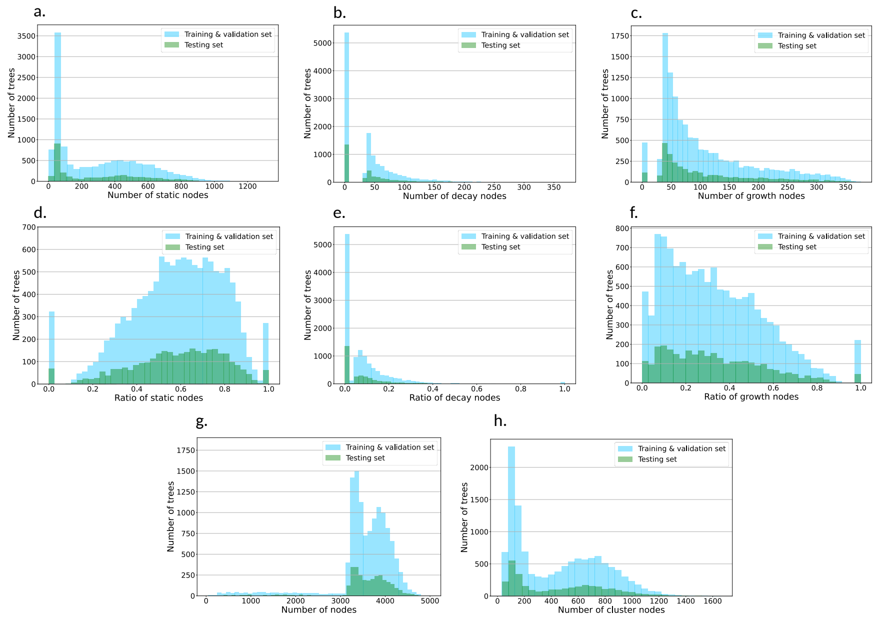

Fig. 3. Statistic information for three different dynamic transmission clusters types on combination of ARI and TB simulations, i.e. static, decay and growth. a, b, and c, are the histogram of number of static, decay and growth nodes among the trees, and plot **d-f** show the distribution of classes' ratio on cluster nodes. Plot **g,** and **h,** show histogram of the number of nodes and number of cluster nodes among trees.

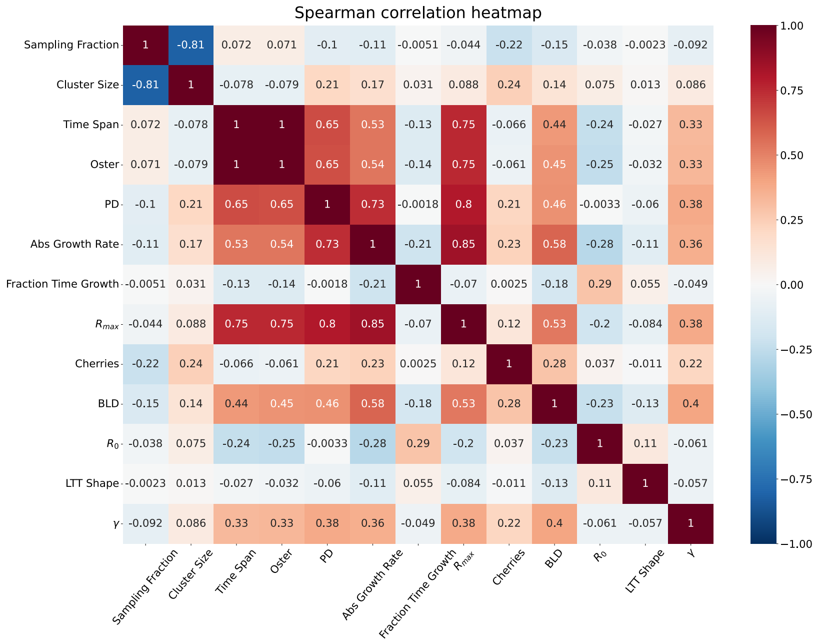

Fig. 4. Spearman's rank correlation on ARI and TB simulations between the ten generalized tree shape metrics and three ground truth cluster characteristics, including sampling fraction, size and time span of the cluster.

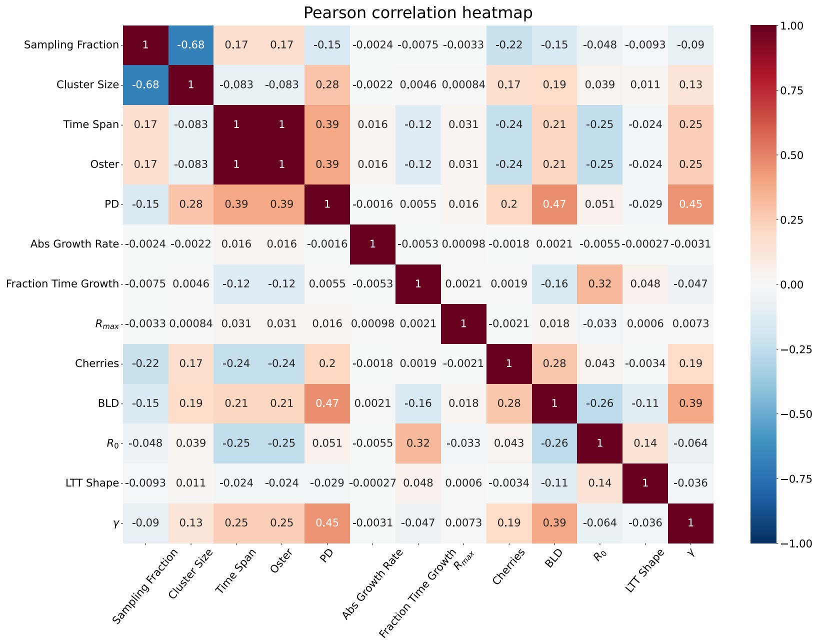

Fig. 5. Pearson correlation on ARI and TB simulations between the ten generalized tree shape metrics and three ground truth cluster characteristics, including sampling fraction, size and time span of the cluster.

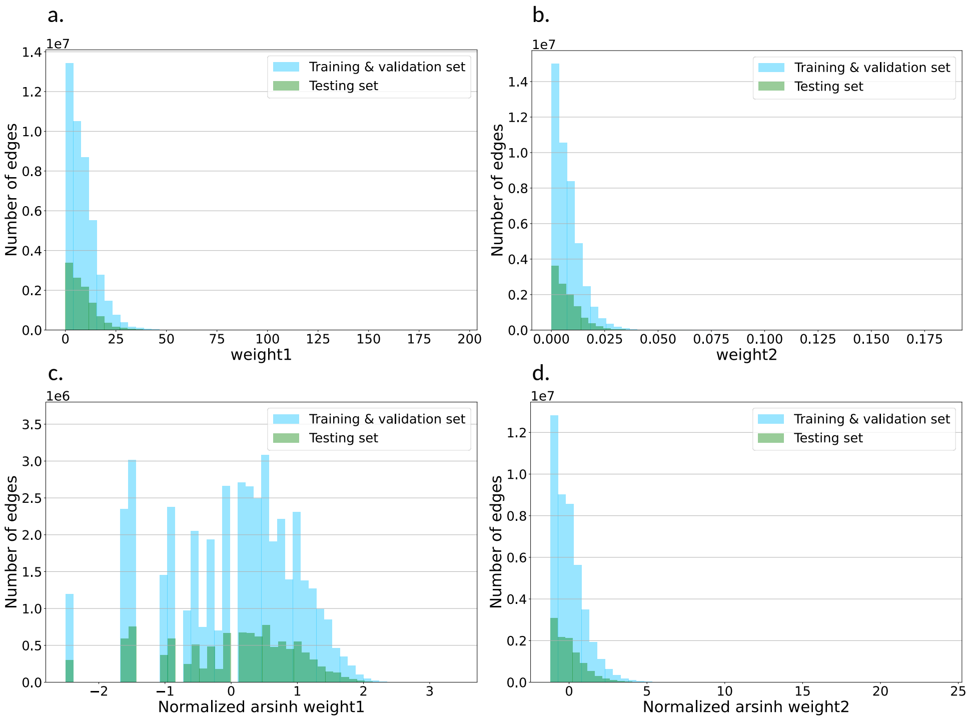

Fig. 6. Distribution of the edge features on ARI and TB simulations. a, and **b,** are distributions of the raw edge features; weight1 and weight2. **c,** and **d,** are distributions of the edge features processed by an ArcSinh transformation and a z-score normalization.

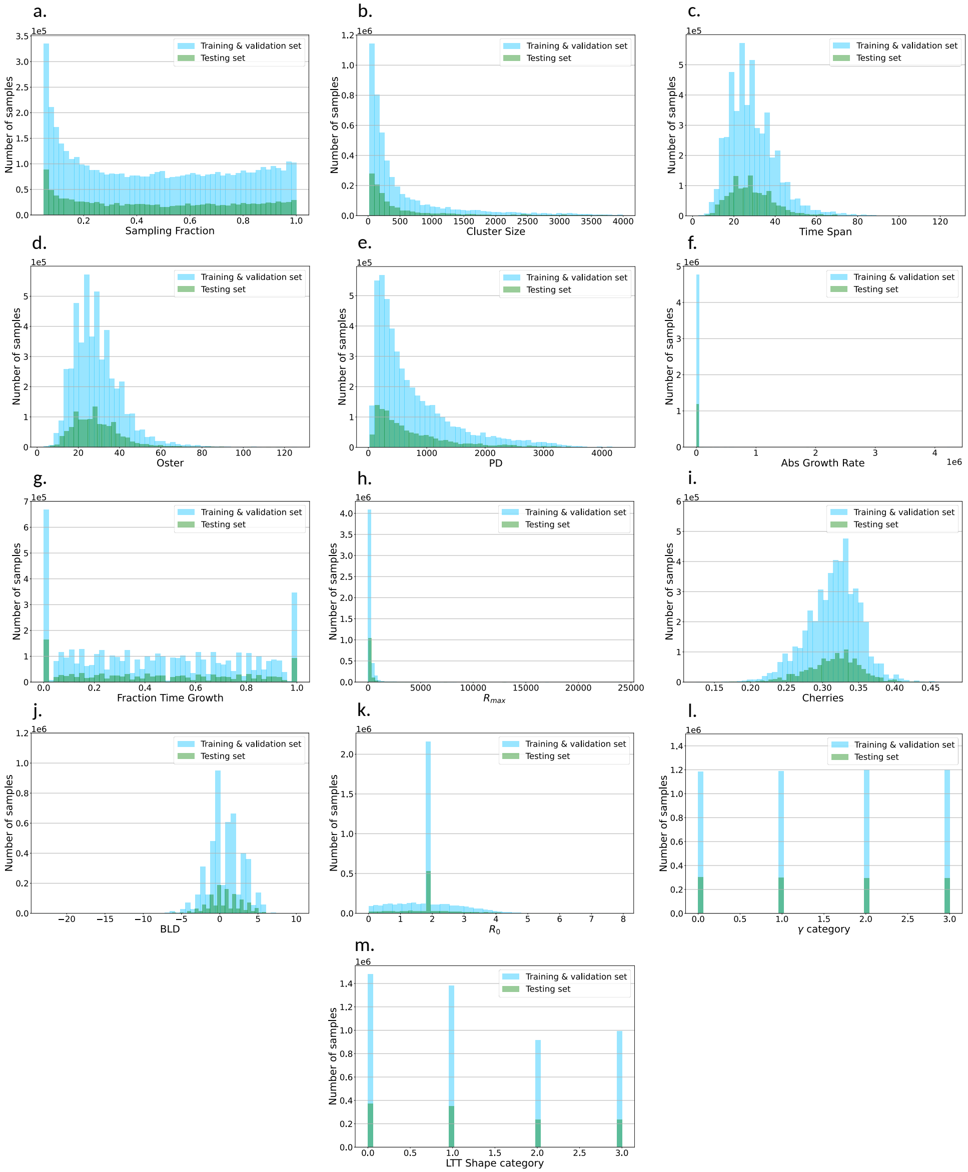

Fig. 7. Distribution of the raw tree shape metrics with numerical values on ARI simulations, including **a,** Sampling Fraction, **b,** Cluster Size, **c,** Time Span, **d,** Oster, **e,** PD, **f,** Absolute Growth Rate, **g,** Fraction Time Growth, **h,** R_{max}, **i,** Cherries, **j,** BLD, **k,** R_0, **l,** γ category and **m,** LTT shape category.

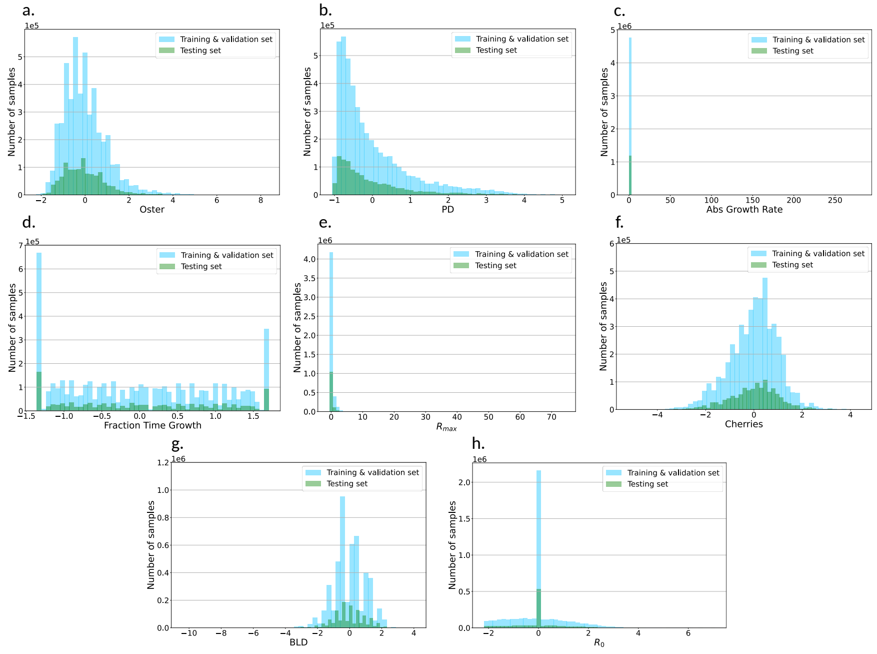

Fig. 8. Distribution of the normalized tree shape metrics with numerical values on ARI simulations, including **a,** Oster, **b,** PD, **c,** Absolute Growth Rate, **d,** Fraction Time Growth, **e,** R_{max}, **f,** Cherries, **g,** BLD, and **h,** R_0.

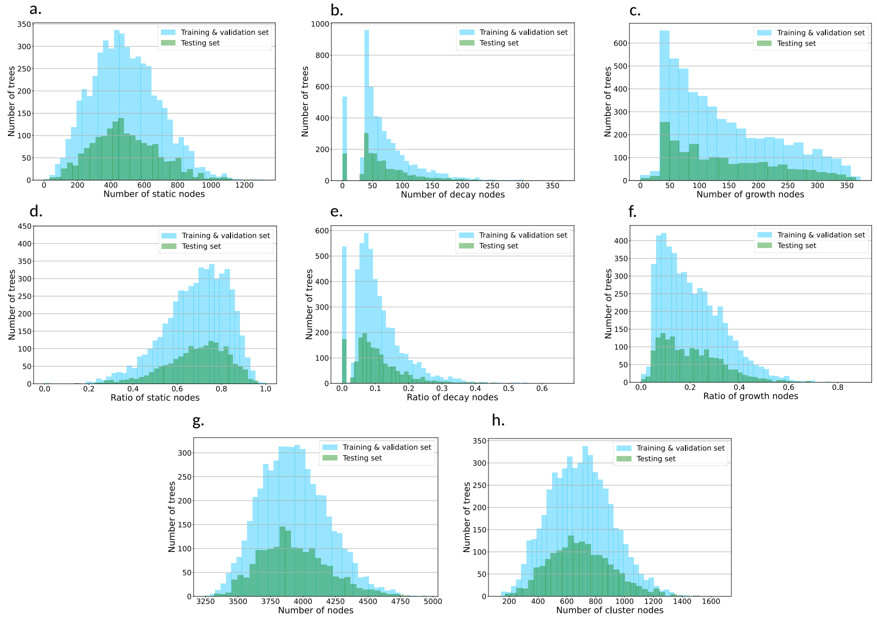

Fig. 9. Statistic information for three different dynamic transmission clusters types on ARI simulations, i.e. static, decay and growth. a, b, and c are the histogram of number of static, decay and growth nodes among the trees, and plot **d-f** show the distribution of classes' ratio on cluster nodes. Plot **g,** and **h,** show histogram of the number of nodes and number of cluster nodes among trees.

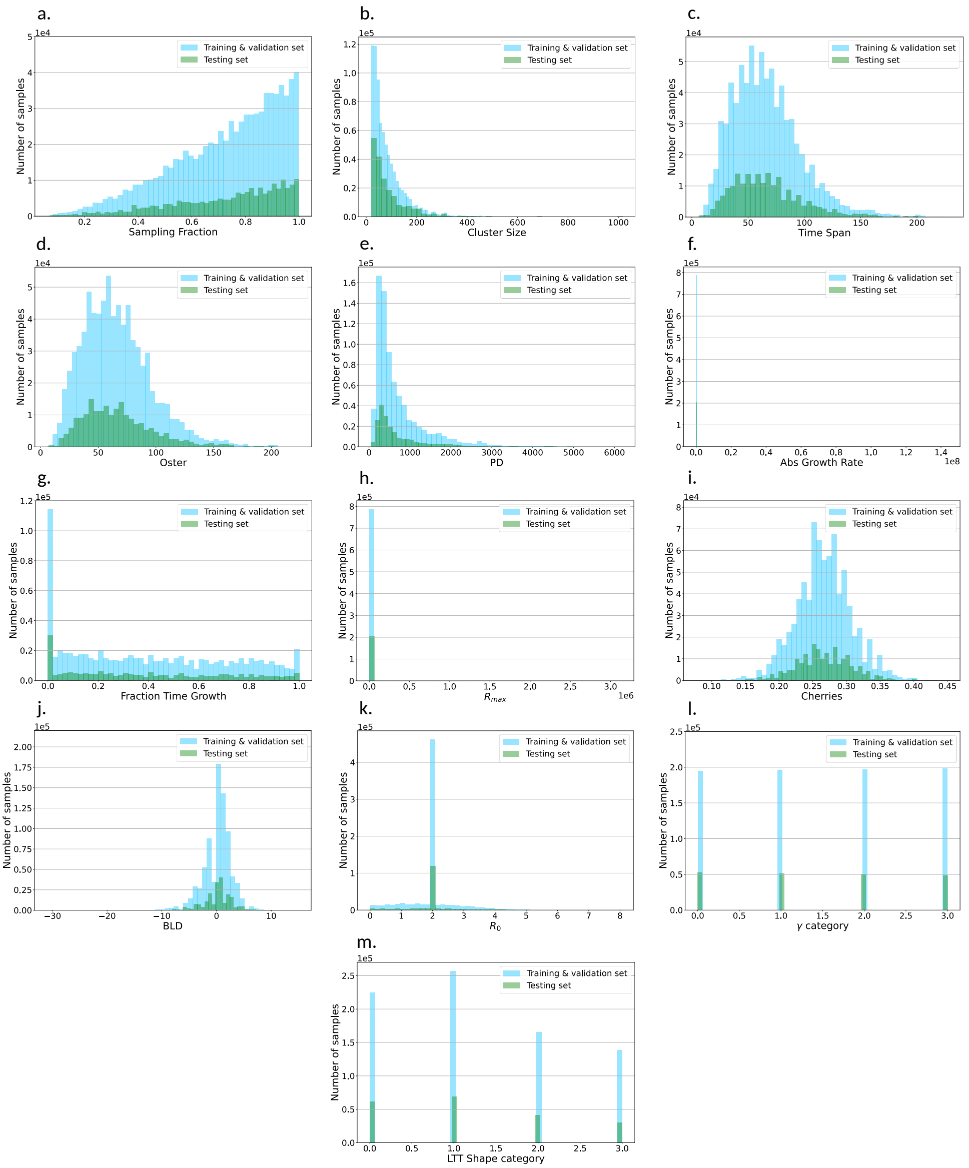

Fig. 10. Distribution of the raw tree shape metrics with numerical values on TB simulations, including **a,** Sampling Fraction, **b,** Cluster Size, **c,** Time Span, **d,** Oster, **e,** PD, **f,** Absolute Growth Rate, **g,** Fraction Time Growth, **h,** R_{max}, **i,** Cherries, **j,** BLD, **k,** R_0, **l,** γ category and **m,** LTT shape category.

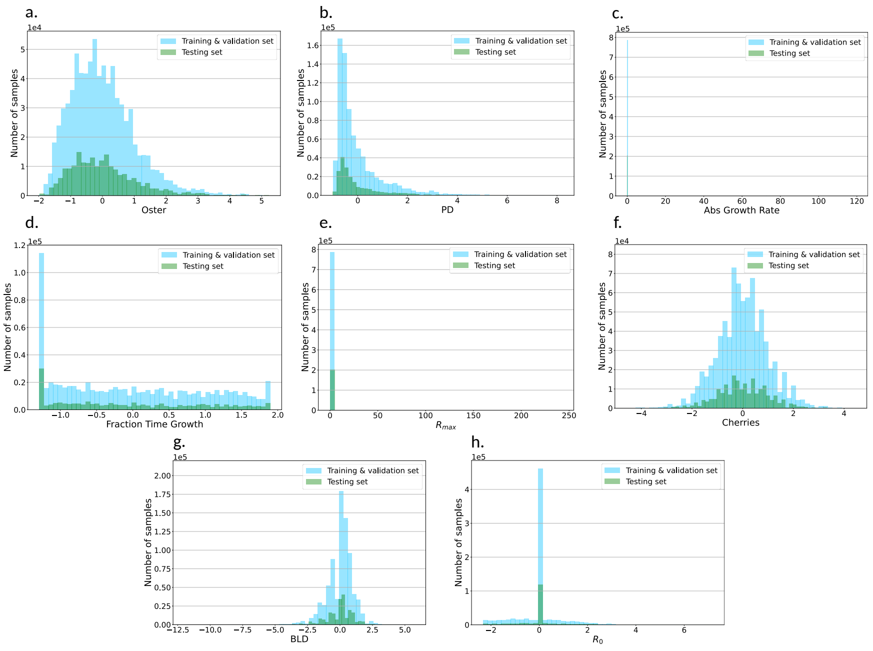

Fig. 11. Distribution of the normalized tree shape meetrics with numerical values on TB simulations, including **a,** Oster, **b,** PD, **c,** Absolute Growth Rate, **d,** Fraction Time Growth, **e,** R_{max}, **f,** Cherries, **g,** BLD, and **h,** R_0.

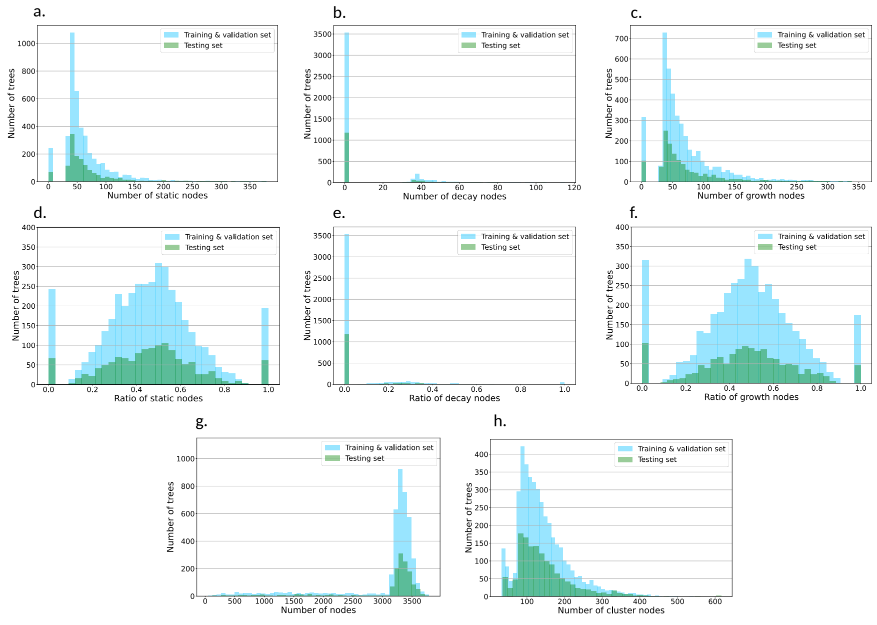

Fig. 12. Statistic information for three different dynamic transmission clusters types on TB simulations, i.e. static, decay and growth. a, b, and c are the histogram of number of static, decay and growth nodes among the trees, and plot **d-f** show the distribution of classes' ratio on cluster nodes. Plot **g,** and **h,** show histogram of the number of nodes and number of cluster nodes among trees.

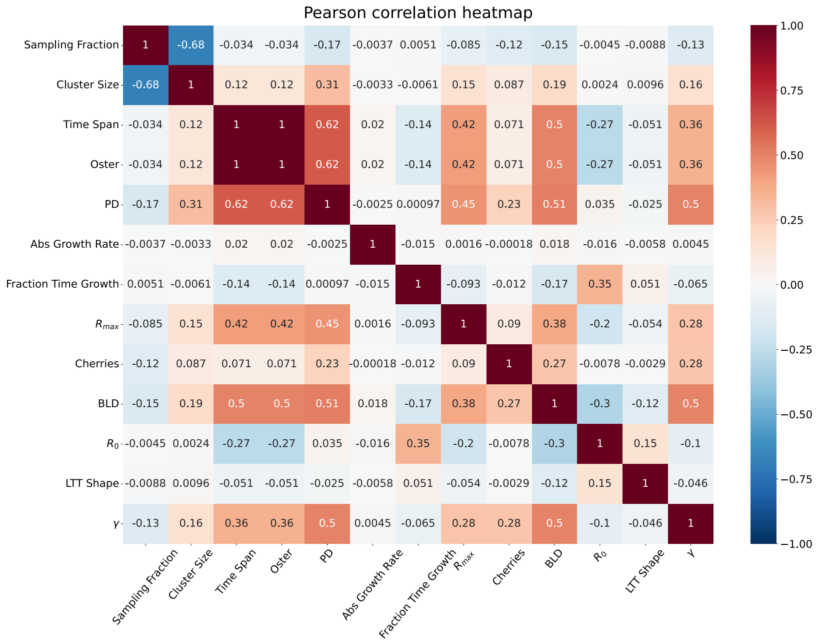

Fig. 13. Pearson correlation on ARI between the ten generalized tree shape metrics and three ground truth cluster characteristics, including sampling fraction, size and time span of the cluster.

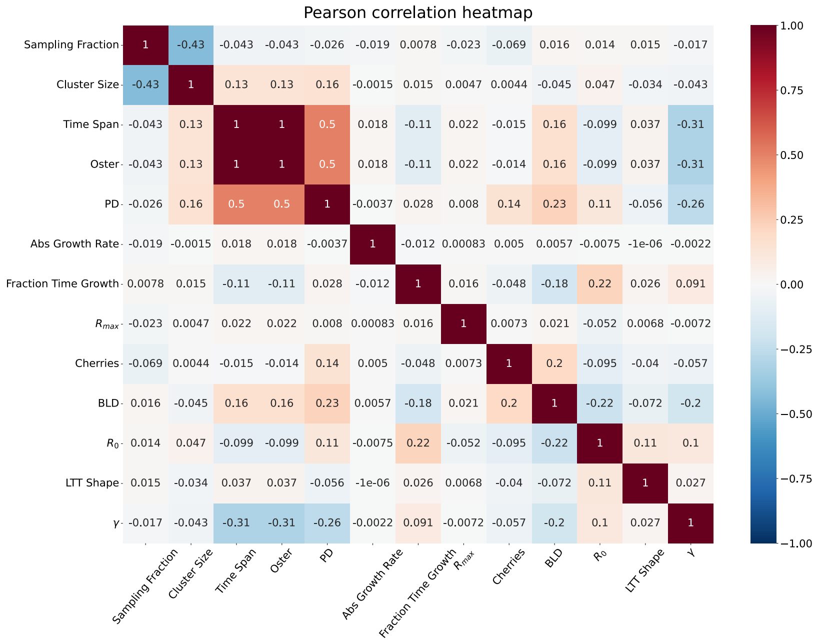

Fig. 14. Pearson correlation on TB between the ten generalized tree shape metrics and three ground truth cluster characteristics, including sampling fraction, size and time span of the cluster.

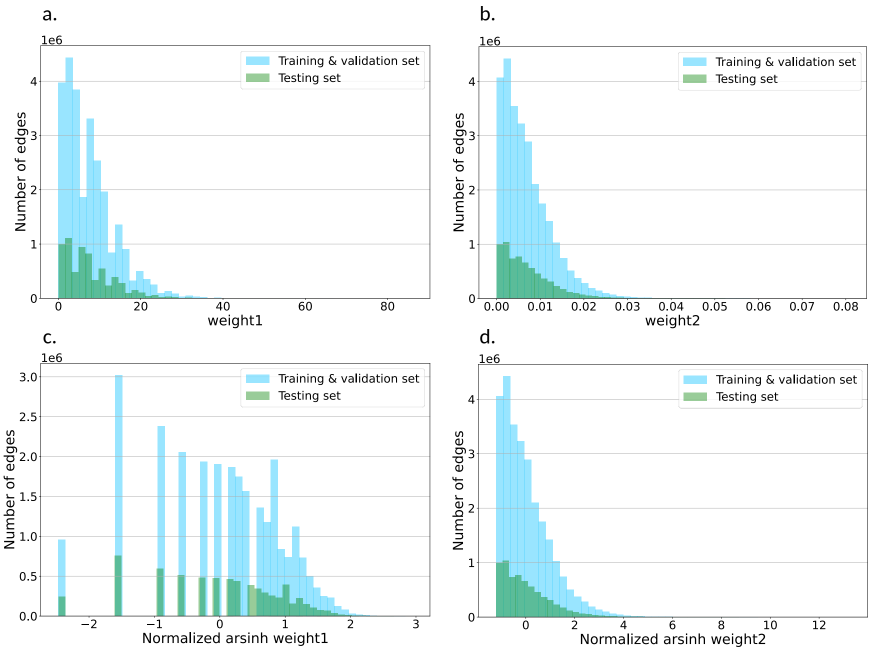

Fig. 15. Distribution of the edge features on ARI simulations. a, and b, are distributions of the raw edge features; weight1 and weight2. **c, and d,** are distributions of the edge features processed by an ArcSinh transformation and a z-score normalization.

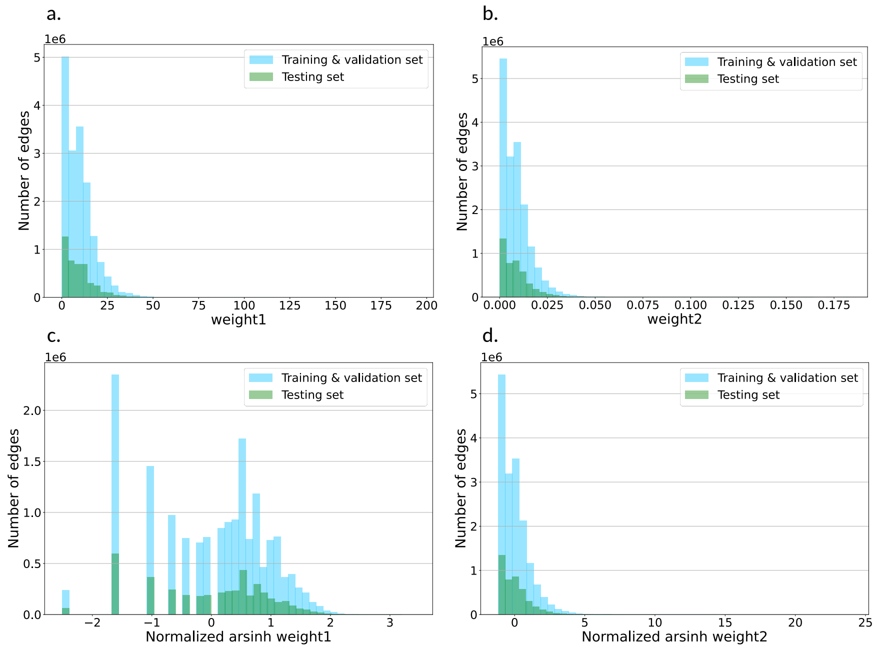

Fig. 16. Distribution of the edge features on TB simulations. a, and b, are distributions of the raw edge features; weight1 and weight2. **c, and d,** are distributions of the edge features processed by an ArcSinh transformation and a z-score normalization.

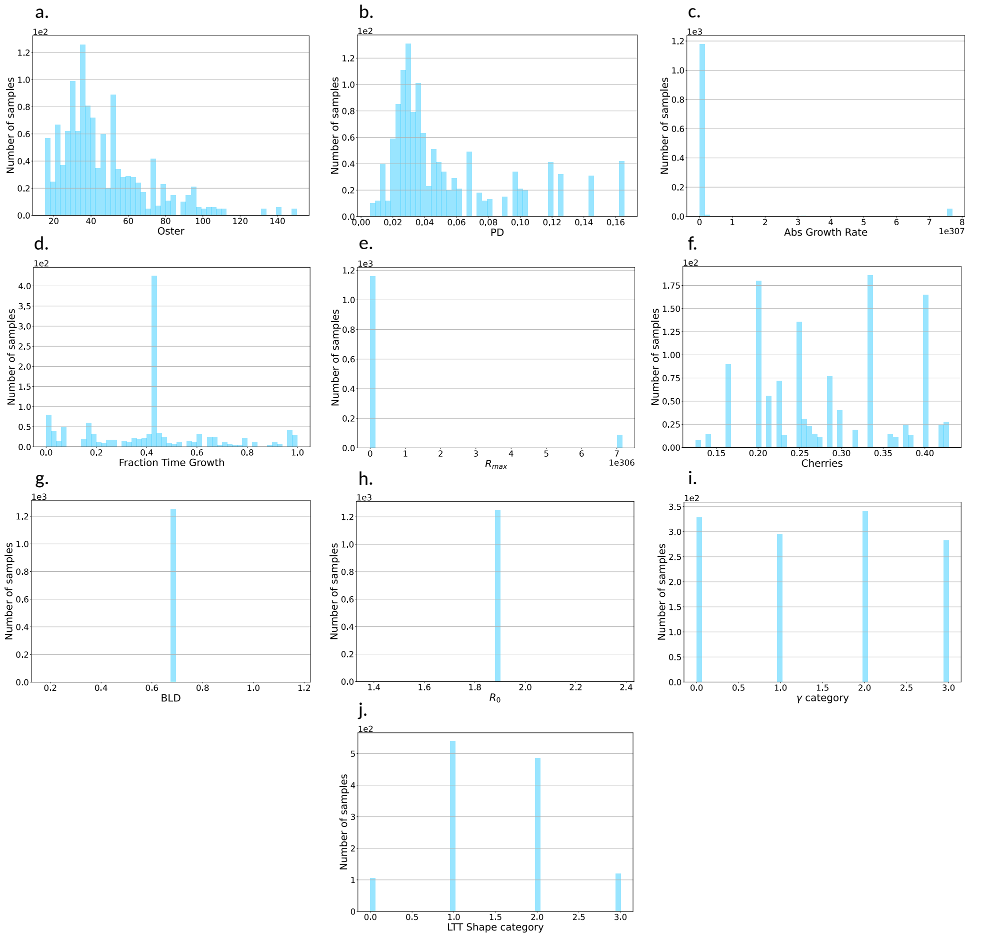

Fig. 17. Distribution of the raw tree shape metrics with numerical values on HIV-1 subtype B outbreak data, including **a,** Oster, **b,** PD, **c,** Absolute Growth Rate, **d,** Fraction Time Growth, **e,** R_{max}, **f,** Cherries, **g,** BLD, **h,** R_0, **i,** γ category and **j,** LTT shape category.

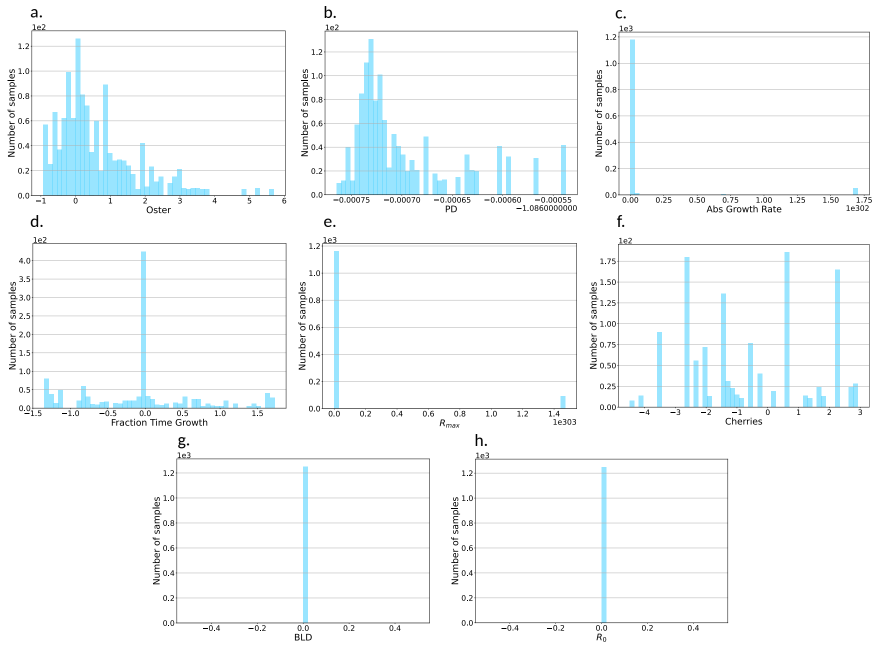

Fig. 18. Distribution of the normalized tree shape metrics with numerical values on HIV-1 subtype B outbreak data, including **a,** Oster, **b,** PD, **c,** Absolute Growth Rate, **d,** Fraction Time Growth, **e,** R_{max}, **f,** Cherries, **g,** BLD, and **h,** R_0.

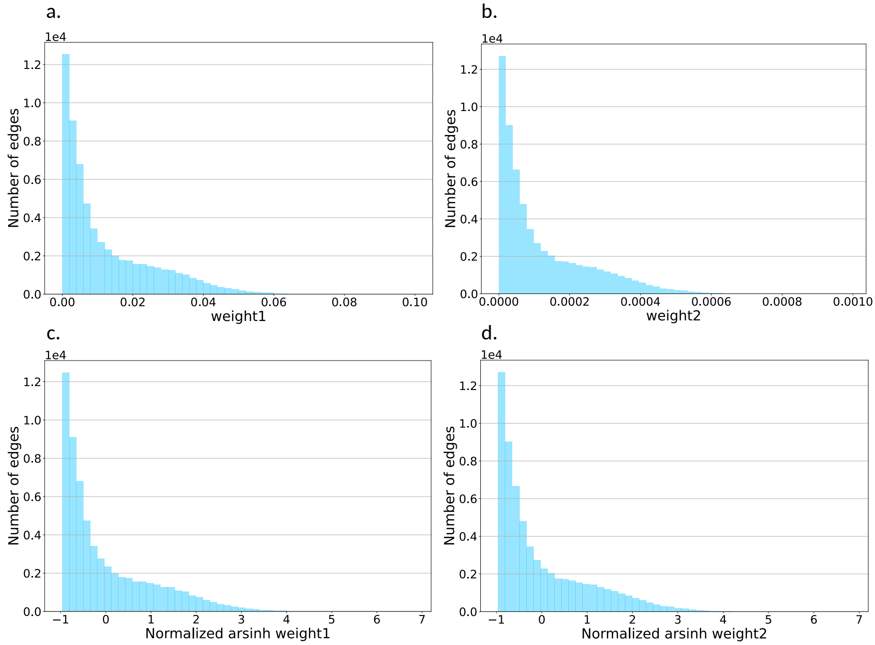

Fig. 19. Distribution of the edge features on HIV-1 subtype B outbreak data. a, and **b,** are distributions of the raw edge features; weight1 and weight2. **c,** and **d,** are distributions of the edge features processed by an ArcSinh transformation and a z-score normalization.

D. Simulation

Simulation of a structured ARI outbreak

For the simulated ARI outbreak, rate of transmission of infection was dependent on the following: 1) the time since initial infection, 2) duration of infection, 3) number contacts, 4) infection rate, and 5) the risk group to which the susceptible individual belongs (Table 3). Incubation periods were specified following infection, during which infected individuals were not permitted to transmit. Individuals were removed from the simulation after an infectious period of ~ 14 days. The number of contacts (N) per infected individual varied according to risk group and number of individuals infected within that group so as to allow for varying dynamics across risk groups. Infection rate was also allowed to vary according to risk group. The rate of infection from a background individual was 1.8×10^{-3} after at least two background individuals had been infected at the start of the simulation. This rate was used to ensure clusters did not exceed in number the background population. The rate of specific infection of dynamic risk groups was two-fold relative to static groups, owing to their lower representation (2:4). Following initiation, transmission was isolated to the risk group (i.e., probability of zero for infecting an individual in another cluster or background population). The number of contacts for groups A-E were picked from a normal distribution with group-specific means and standard deviation, representing clusters for which the rate of secondary infection (R_e) remained steady, or static. The number of contacts for F and G, however, were derived from the following linear function:

$$N = rh + N_0, \tag{1}$$

where N_0 is the initial number of contacts, and r is the rate of change dependent on the current number of actively infected hosts in the simulation (h). Cluster F was considered to be experiencing an increasing rate of growth in transmission over time, whereas G was considered to be decaying over time (Table 3). Multiple static clusters were also incorporated with varying contact parameters in order to determine indirectly the relationship of these parameters with branching patterns and thus influence on cluster classification. Each of a total of 10,000 simulations was run for 365 days or until a total of 10,000 hosts were infected.

Table 3. Simulation information for each outbreak and risk group.

Outbreak scenario	Risk group	Contact parameters[1]	Mean P(T)[2]	Mean R_0
ARI	A	$\sim \mathcal{N}(16, 1)$	0.015	2.2
ARI	B	$\sim \mathcal{N}(4, 1)$	0.09	3.2
ARI	C	$\sim \mathcal{N}(4, 1)$	0.11	3.9
ARI	D	$\sim \mathcal{N}(6, 1)$	0.09	4.9
ARI	E	$\sim \mathcal{N}(4, 1)$	0.12	4.3
ARI	F	$N_0 \sim \mathcal{N}(4,\ 1)$, $r \sim \mathcal{N}(0.018,\ 0.009)$	0.11	0.8
ARI	G	$N_0 \sim \mathcal{N}(6,\ 1)$, $r \sim \mathcal{N}(0.18,\ 0.09)$	0.11	9.8
TB/HIV	A	$\sim \mathcal{N}(20, 5)$	0.025	1.5
TB/HIV	B	$\sim \mathcal{N}(20, 5)$	0.015	0.9
TB/HIV	F	$N_0 \sim \mathcal{N}(15,\ 1)$, $r \sim \mathcal{N}(0.18,\ 0.09)$	0.015	0.7
TB/HIV	G	$N_0 \sim \mathcal{N}(20,\ 1)$, $r \sim \mathcal{N}(1.8,\ 0.9)$	0.025	1.5

[1] Single values represent mean number of contacts for static clusters.
[2] Probability of transmission

Simulation of a structured TB/HIV outbreak

A previously described co-infection outbreak model describing the impacts of HIV infection on the spread of TB was adapted herein from Goldstein *et al.* Goldstein et al. (2022). Briefly, the mean incubation period, though more appropriately referred to as the latent period for TB, was 9 months among hosts that became infectious, and the mean infectious period was 3 months. The number of contacts per infected individual varied according to risk group and number of individuals infected within that group so as to allow for varying dynamics across risk groups. Infection rate was also allowed to vary according to risk group, and, unlike the ARI outbreak, transmission to individuals outside of each risk group was permitted. The rate of infection from a background individual was 1.8×10^{-2} after at least two background individuals had been infected at the start of the simulation, and the relative rate of infection of an individual belonging to a particular risk group was dependent on the risk group - two main risk groups were included, representing TB-infected individuals living with or without HIV. These groups were further split into static and dynamic transmitting groups. Based on a reported average of 53% percent of infection recipients harboring HIV Goldstein et al. (2022), infection rate for HIV risk groups A and D were $0.53/2 = 26.5\%$. Remaining non-HIV-infected risk groups B and E comprised the remaining 47% (equally represented). The number of contacts for groups A and B were picked from a normal distribution with group-specific means and standard deviation, representing clusters for which the rate of secondary infection (R_e) remained steady, or static. The number of contacts for F and G, however, were derived from the linear equation as in Eq. 1 and are also described in Table 3. Group F was considered to be experiencing an increasing rate of growth in transmission over time, whereas G was considered to be decaying over time (Table 3). Group B was thus considered to exhibit static transmission. Each of a total of $10,000$ simulations was run for 8 years or until a total of $10,000$ hosts were infected.

Reconstruction of sampled transmission tree from simulated outbreaks

Recipients were only sampled during their infectious periods, with the sampling time equally likely at any point in this time frame. Only simulations where at least 50 individuals were infected were accepted. One representative clade within the background population was chosen at random from the corresponding internal nodes and maintained all individuals (5-100), representing true clusters of direct transmission, or transmission chains. Remaining risk groups were sampled randomly, ranging in frequency from 20-100% of the original cluster population. The background population was also downsampled at a frequency of 20%, representing a more realistic surveillance scenario. Hosts not included within this sample were pruned from the full tree to obtain the final set of simulated trees used for tree metric calculation and deep learning models. A relaxed molecular clock (evolutionary rate in time) was assumed, and branch lengths were scaled in time (substitutions/site/year) using a uniform distribution ($\sim \mathcal{U}(8 \times 10^{-4}, 0.001)$), allowing for both genetic distance and time to be used as distinct weights in the neural network models.

E. Sampling bias influence on dynamic inference

Sample size is the most basic of epidemiological attributes that can wreak havoc on quantitative estimates, with smaller sampling sizes providing less reliable estimates owing to the limited amount of information represented. Additionally, considering a large fraction of the tree shape metrics rely on branch lengths scaled in time, the total time during which a cluster has taken place (calculated using branch lengths) may similarly influence the uncertainty of the estimate. Whereas some level of correlation is expected between cluster size, time span, and true cluster dynamics (e.g., growing clusters should intuitively be expected to be larger in size as well as time span), this relationship breaks down under two scenarios - firstly, a cluster characterized by a greater transmission potential might not emerge until the end of the simulation, which is terminated conditionally on the total number of individuals infected ($10,000$) or total days occurring (365). The end of the simulation can also be thought of as the most recent collection date prior to data analysis in a real-life study. Secondly, a small cluster size may actually represent a large cluster sampled at low frequency (included in the simulation), as is often a realistic scenario in epidemiology, owing to under-represented populations in health care or undetected transmission among asymptomatic individuals, for example. Hence, if the performance of *DeepDynaTree* were to be heavily influenced by sampling frequency, increased efforts in field investigation would lend increasing confidence in the classification

of a cluster. Sampling fraction also has another potential influence on the use of tree shape metrics in predicting dynamics, as the estimate of N_e described above assumes a relatively small fraction of the total population has been sampled. As this fraction approximates the census, or total population size, external branch lengths are exaggerated, and the tree gives the appearance of inflated growth (Wakeley and Takahashi, 2003). Hence, while sufficient, unbiased sampling can increase the sample size and thus informativeness of the data, over-sampling (as with a fully sampled transmission chain) may result in falsely classified clusters as epicenters of transmission.

F. Baseline approaches

Phylogenetic tree representations and notations

We represent a phylogenetic tree using a tree structured graph notation $G = (V, E)$, where $V = \{v_1, ..., v_N\}$ is a set of N nodes, and $e_{ij} \in E$ is an edge from node v_i to node v_j. $\mathbf{v}_i$ and $\mathbf{e}_{ij}$ denote the features of node v_i and edge e_{ij}. An adjacency matrix denoted as $\mathbf{A} \in \mathbb{R}^{N\times N}$ represents the connectivity of various nodes, and the neighborhood set of the node v_i is represented as $\mathcal{N}(v_i)$. The key annotations are listed in Table 4, where bold uppercase characters are used to denote the matrices and bold lowercase characters denote the vectors.

Node-based methods using generalized tree shape metrics

We first attempted to assess the value of a subset of relevant tree shape metrics (described in more detail in Supplementary Section A) in predicting the dynamics of simulated transmission clusters belonging to differing risk groups. Each node within a cluster was represented by 10 such features, and the full data set was represented in a common tabular data format, comprising nodes (rows) with the same set of features (columns). Different machine and deep learning algorithms were developed to predict the nodes' dynamic characteristic label (static, growing, or decaying), heretofore referred to as the node-based methods because they focus on node features only. The following two subsections describe the developed algorithms in details.

We investigated three broadly-used machine learning methods ranging from logistic regression (LR) (Hosmer Jr et al., 2013) to two strong and robust ensemble methods - random forest (RF) (Breiman, 2001) and Extreme Gradient Boosting (XGboost) (Chen and Guestrin, 2016). LR served as the simplest baseline method, which fits a generalized linear model with $60 = 10\times3\times2$ parameters in our experiment to minimize the residual sum of squares between node labels and the linear approximations. To deal with the multiclass case, a multinomial logistic regression was utilized to train the model, and a L2 regularization term with factor 0.001 was applied to prevent over-fitting. RF is a strong and robust bagging ensemble method, which develops diverse decision trees on the bootstrap-sampled subsets of the original training set and then aggregates their outputs as a final prediction (Breiman, 2001). In our experiment, we assembled 70 tree classifiers, and in order to control over-fitting, the max depth of each tree was constrained as 5, and the minimum

Table 4. Commonly used notations in this paper.

Notations	Descriptions
$\mathbb{R}^F$	F-dimensional Euclidean space
$G = (V, E)$	A graph
$V = \{v_1, ..., v_N\}$	The set of N nodes
$e_{ij} \in E$	An edge e_{ij} from node v_i to v_j
$\mathbf{v}_i$	The features of node v_i
$\mathbf{e}_{ij}$	The features of edge e_{ij}
$\mathbf{A}$	The adjacency matrix
$\mathcal{N}(v_i)$	The neighbors of v_i
**W**, **U**, **b**	The learnable parameters
$\sigma(\cdot)$	The activation function
$[\cdot, \cdot]$	The concatenation of vectors

number of samples required to be at a leaf node was set as 12. Another utilized machine learning algorithm, XGBoost, is one of the most popular and effective Gradient Boosting Decision Tree (GBDT) algorithms. Unlike RF, XGBoost utilizes the boosting ensemble method, which fits multiple base models sequentially such that the training of the current base model at a given step depends on how the previous base models fit the training set (Chen and Guestrin, 2016). We selected gradient-boosted decision trees as the base model, and to again avoid over-fitting, we slowed the boost learning step by a shrinkage factor 0.07, and the training samples were sub-sampled by 7.79% in every boosting iteration. To reduce the model complexity, the weight for L2 regularization was set as 1, and the depth of trees was constrained to be 20. Both RF and XGBoost models have been successfully applied in many machine learning tasks and achieved state-of-the-art results, especially for input data with tabular format (Shwartz-Ziv and Armon, 2021).

We also investigated several deep learning-based approaches (i.e., multilayer perceptron (MLP) (Haykin, 1994), DeepSet (Zaheer et al., 2017), SetTransformer (Lee et al., 2019), and TabNet (Arık and Pfister, 2020)). A frequent and straightforward choice of neural network architecture to learn from a set of independent features, MLP was composed in this study of 7 fully connected layers, containing 128 hidden units for each layer, followed by batch normalization layers. The Leaky Rectified Linear Unit (LeakyReLU) was then used as the nonlinear active function. A more complex deep learning architecture for tabular learning, TabNet uses a sequential attention mechanism to select a subset of features to process at each decision step, enabling better interpretability and learning capacity. For TabNet, the dimensions of both prediction layer and attention layer were set to 64. The number of successive steps was set to 10, and each step included 2 independent Gated Linear Unit (GLU) layers and 2 shared GLU layers. The scaling factor for attention updates was set to 2, and momentum for batch normalization 0.02. The masking function sparsemax (Arık and Pfister, 2020) was used for feature selection.

Besides treating the nodes' features as tabular data, we also explored another view of the feature format, which considers them as set-structured data. Unlike the fixed dimensional vectors, the output label for the entire set should be invariant to the permutation of set elements (that is, the order of input features). Such problems are widespread, such as 3-dimensional shape recognition from point clouds, wherein the shape label is invariant to the order of points. The models used to address them should be *permutation invariant*, which means the predictions should not depend on the order of elements in the feature set. DeepSets (Zaheer et al., 2017) is one of the pioneering works used in solving set-input problems. In the model, each feature of the set is first individually embedded by a neural network, and then a permutation invariant operation (e.g., sum) is applied to aggregate all embeddings, or output of inner layers in the neural network. The final output is generated by applying another neural network on the aggregation in the same manner as in any deep network (e.g., fully connected layers, non-linear active function, etc.). In our setting, both encoder and decoder included 4 fully connected layers and each layer included 256 hidden units. The permutation invariant operation was the average over embedded features of encoder's output. SetTransformers (Lee et al., 2019) is another popular permutation invariant method, which improves on DeepSets by using a self-attention mechanism to process every feature in the set. SetTransformers enables discovery and modeling of the potential interactions among each node's metric features. In our experiment, the encoder included 3 Induced Set Attention Blocks (ISAB) modules with 2 heads and 64 hidden units. The decoder consisted of 1 Pooling by Multihead Attention (PMA) module and 2 Set Attention Blocks (SAB) with 4 heads.

DeepDynaTree - A phylogenetic-informed approach

Unlike the previously mentioned approaches, our designed *DeepDynaTree* platform is phylogenetically informed, which means it not only utilizes the tree shape metrics used in the node-based models described above but also the underlying topological and branch length information within the tree itself. To achieve this, we proposed the use of a GNN to learn from the raw phylogenetic tree directly and provide the node-wise classification. GNN is a type of deep learning model designed for addressing graph-related tasks. A typical GNN usually consists of several trainable layers and operations (e.g., propagation and aggregation, updating, readout or pooling, etc.) The propagation operation is used to communicate information between nodes or edges based on an information diffusion mechanism so that the aggregated information can capture both nodes' or edges' features and phylogenetic information. Then GNN updates nodes' states by considering

the aggregated neighborhoods' information, or connected edges', information recurrently. Similarly, the edges' feature can also be updated by exchanging the connected nodes' information. When the subgraph's or graph's high-level representation is required, a readout operation is used to extract information from nodes and edges, and common techniques for readout, including sum, average and max pooling. These layers are usually stacked repeatedly, and, with the increase of the number of layers, the receptive field size of a node will correspondingly increase, resulting in more high-level and informative representations. In *DeepDynaTree*, a phylogenetic tree is considered a static bidirectional homogeneous graph, wherein each internal node represents the most recent common ancestor of the two lineages descended from that node and an edge between two nodes represents their evolutionary and/or temporal distance. The dynamic prediction of transmission clusters is formulated as a node-level classification task on the graph. In other words, each risk group node is classified as belonging to the category of static, decay or growth. It is worth noting that we also explored to formulate a phylogenetic tree as a directed graph, which allows to propagate the messages in one direction only, i.e., downwards from tree root to leaves. However, the performance of this approach was not satisfactory compared to the bidirectional message passing method. We first assessed three popular GNN variants - Graph Convolutional Network (GCN) (Kipf and Welling, 2017), Graph Attention Network (GAT) (Veličković et al., 2018), and Graph Isomorphism Network (GIN) (Xu et al., 2019). In contrast with the node-based approaches, these methods consider the underlying relationship between different cluster nodes, instead of viewing each node individually. However, it is worth noting that these GNN variants can only utilize the basic topological information in the phylogenetic tree (i.e., connectivity) without adequately utilizing the branch length information, representing the genetic distance and/or evolutionary time separating individual nodes within the tree. For example, for GCN, the weights for aggregating neighbors are defined by the tree topological structure (i.e., an adjacency matrix). Similarly, in GAT, the attention coefficient is calculated based on the neighbors' features and is masked as zero when there exists no direct connection between any two nodes.

GCN is a typical spectral network, which utilizes a first-order approximation of spectral graph convolution with localized filters on the nodes of an input graph (Kipf and Welling, 2017). Formally, a layer-wise propagation rule is defined as:

$$\begin{aligned} \mathbf{V}^{l+1} &= \sigma(\widetilde{\mathbf{D}}^{-\frac{1}{2}}\widetilde{\mathbf{A}}\widetilde{\mathbf{D}}^{-\frac{1}{2}}\mathbf{V}^{l}\mathbf{W}^{l}) \\ \textit{where} \quad \widetilde{\mathbf{A}} &= \mathbf{A}+\mathbf{I}_N, \widetilde{\mathbf{D}}_{ii} = \sum_{j=1}^{N}\widetilde{\mathbf{A}}_{ij} \end{aligned} \tag{2}$$

where $\mathbf{V}^l \in \mathbb{R}^{N\times C}$ and $\mathbf{V}^{l+1} \in \mathbb{R}^{N\times F}$ respectively represent the input and output node features for l-th layer with C and F dimensions. $\mathbf{V}^0$ corresponds the original generalized tree shape metrics of all N nodes. $\widetilde{\mathbf{D}}$ is a diagonal matrix of node degrees and $\mathbf{I}_N$ is an identity matrix.

We stacked 11 layers of the spectral graph convolution, and each layer contained 128 hidden units. Unlike the original semi-supervised training scheme proposed in (Kipf and Welling, 2017), in our experimental setting, we adopted the supervised training manner wherein all the risk group nodes in the test set did not have any label information, and nodes in the training and validation subsets were all labeled.

The second model, GAT, was originally proposed by Velickovic *et al.* (Veličković et al., 2018), which introduces the self-attention mechanism into the graph learning, by which the node state updates depend on the attention coefficients over its neighbors. Specifically, the attention coefficients are calculated as:

$$\begin{aligned} \alpha_{ij}^{(k)l} &= \frac{\exp(\text{LeakyReLU}(a_{ij}^{(k)l}))}{\sum_{n\in\mathcal{N}(i)}\exp(\text{LeakyReLU}(a_{in}^{(k)l}))} \\ \textit{where} \quad a_{ij}^{(k)l} &= \mathbf{a}^{(k)l^T}[\mathbf{W}^{(k)l}\mathbf{v}_i^l, \mathbf{W}^{(k)l}\mathbf{v}_j^l] \\ \mathbf{v}_i^{l+1} &= \|_{k=1}^{K}\,\sigma(\sum_{j\in\mathcal{N}(i)}\alpha_{ij}^{(k)l}\mathbf{W}^{(k)l}\mathbf{v}_j^l) \end{aligned} \tag{3}$$

where $a_{ij}^{(k)l}$ is a scalar representing the importance of node v_j to node v_i for l-th layer and k-th head, and the attention coefficient $\alpha_{ij}^{(k)l}$ regarding to node v_i is normalized with LeakyReLU non-linear

function and softmax function. $\mathbf{v}_i^l \in \mathbb{R}^F$ represents the features of node v_i, where $F = K * H$ for K heads and H output dimensions of each head, and $\mathbf{a} \in \mathbb{R}^{2H}$ is learnable weight vector. $||$ here stands for the operation concatenating multiple vectors. In our setting, we stacked 8 graph attention layers, the multi-head attention of each layer was applied with the number of 3, and each head contained 64 hidden units.

Xu et al. (Xu et al., 2019) proposed the GIN variant, which supposedly achieves the maximum discriminative power among GNN. It uses a multilayer perceptron (MLP) model to update the node features as:

$$\mathbf{v}_i^{l+1} = \mathrm{MLP}^{l+1}\left(\left(1 + \epsilon^l\right) \cdot \mathbf{v}_i^l + \sum_{j \in \mathcal{N}(i)} \mathbf{v}_j^l\right) \tag{4}$$

where ϵ is either a learnable parameter or a fixed scalar. Different from generating whole graph embedding as proposed in (Xu et al., 2019), here we directly apply fully-connected layers on nodes' feaures in each layer and generate the final prediction score by summation:

$$\mathbf{s}_i = \sum_{l=1}^{L} \left(\mathbf{W}^l \mathbf{v}_i^l + \mathbf{b}^l\right) \tag{5}$$

where $\mathbf{s}_i \in \mathbb{R}^C$ represents the predicted score of C classes. L is the number of GIN layers. In our implementation, ϵ was set as a learnable parameter. we stacked 10 GIN layers, the MLP of each layer was applied with 5 layers, and each layer contained 64 hidden units.

G. Additional experimental results

Figure 20 illustrates the performance comparison of all the benchmarking algorithms using the averaged receiver operator characteristic curves, confusion matrices, and permutation feature importance results. Figure 21 shows the permutation feature importance results of *PDGLSTM*, and the absolute reduction of balanced accuracy on the testing dataset was used as the measurement. Figure 22 shows the reduction of model performance with permutating two features at the same time. Limitation analysis on *PDGLSTM*, i.e., the predicting accuracy distribution over ground truth cluster characteristics, is provided in Figure 23. Figure 24 illustrates how to construct a dual graph from a phylogenetic tree.

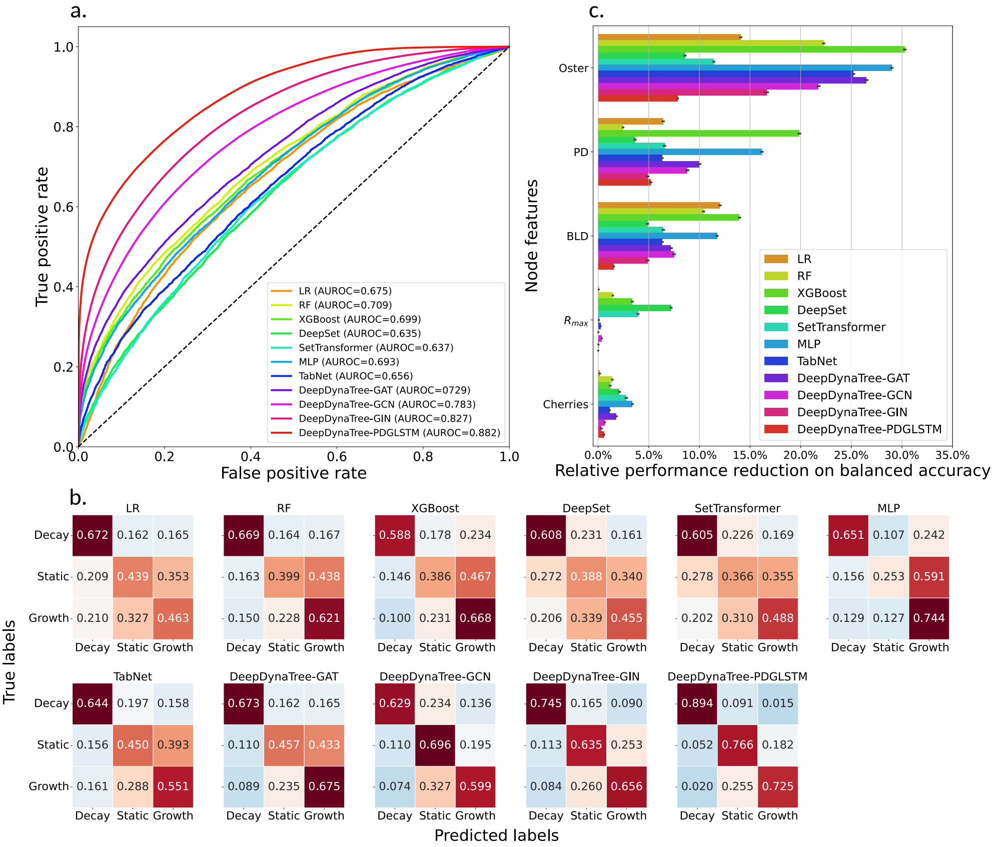

Fig. 20. Figurative performance comparison of all classifiers and permutation feature importance results. a, Comparison of various classifiers, shown on a equally averaged receiver operator characteristic curve, with AUC indicated for each classifier. **b,** Comparison of various classifiers, shown on confusion matrices, and elements are row-wise normalized by class support size. **c,** Relative permutation feature importance results measured by the balanced accuracy. x-axis represents the relative model performance reduction in percentage compared to the non-permutated model and y-axis represents the top-5 most important generalized tree shape metric features. The features are ranked based on the average relative reduction over all the models, and their relative reductions' mean and standard deviation were measured over 50 runs.

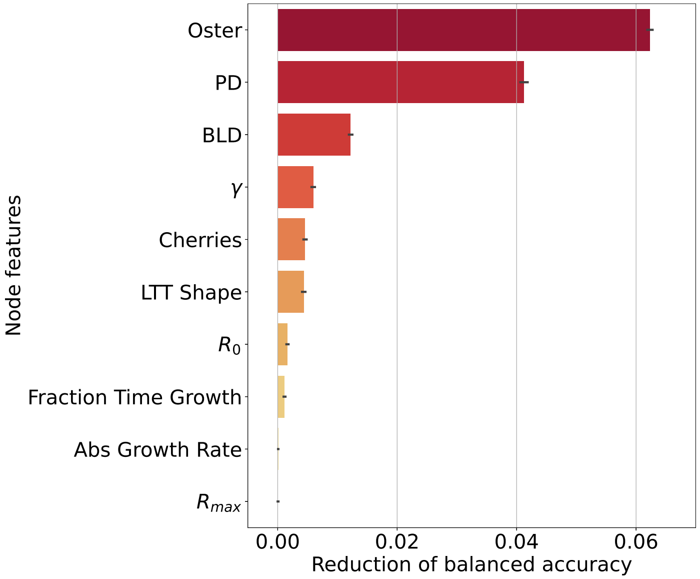

Fig. 21. Permutation feature importance results for *DeepDynaTree-PDGLSTM*. Features were ranked by the permutation importance scores. The mean and variance of each feature's permutation importance were measured over 50 runs.

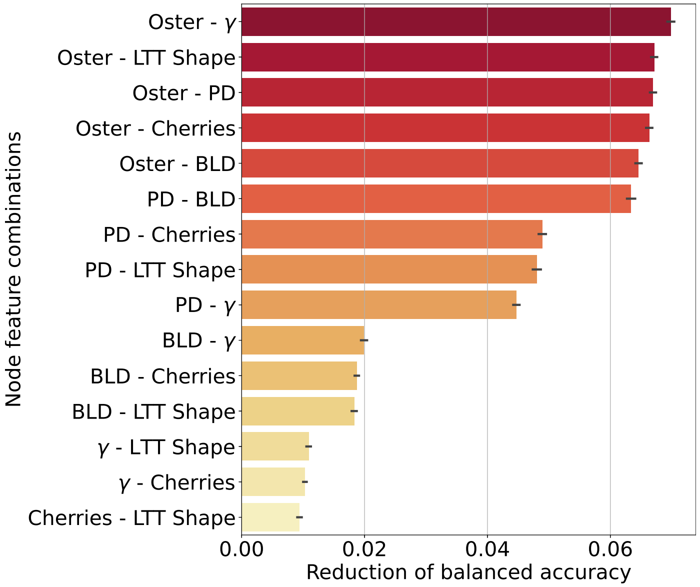

Fig. 22. Permutation importance of feature combinations for *DeepDynaTree-PDGLSTM*. Top-6 most important features in Figure 21 were selected in this test. The mean and variance of each feature's permutation importance were measured over 50 runs.

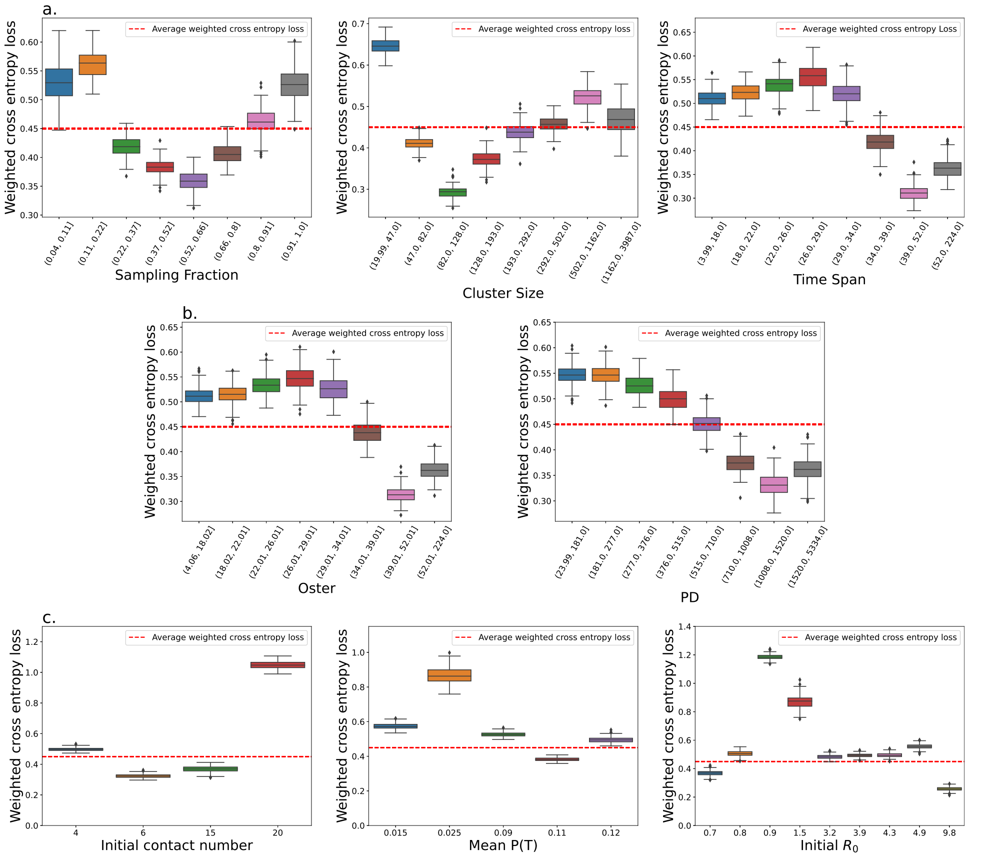

Fig. 23. Sensitivity of the *DeepDynaTree PDGLSTM* model to important features and cluster attributes. Each plot shows the weighted cross entropy value for each interval or category. Binned intervals for quantitative data were generated using eight quantiles. Red dashed lines show the weighted cross entropy loss and 95% confidence intervals for the test and provide a threshold for model performance sensitivity. The boundaries of confidential intervals are close to each other so the intervals are unclear. **a,** All ground truth cluster attributes. **b,** Primary contributing tree metric features. **c,** Varying risk groups within the static category of transmission clusters (see Table 3).

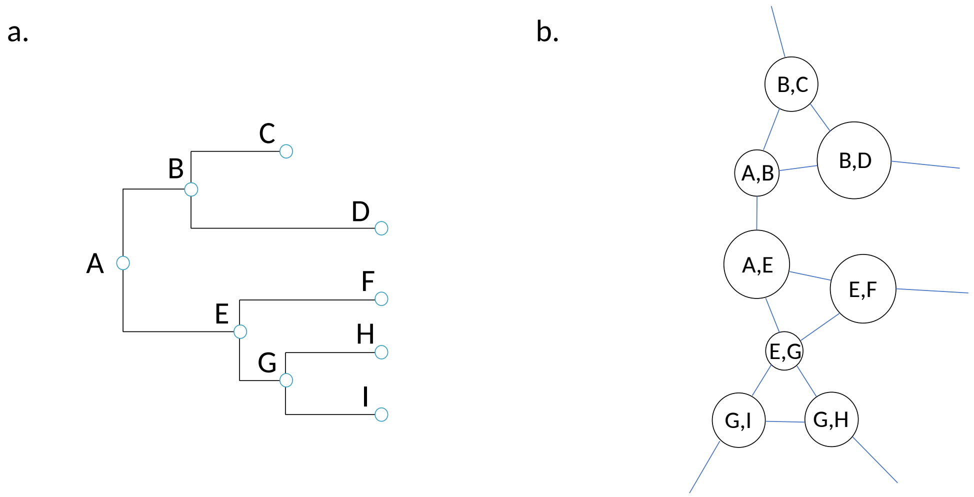

Fig. 24. An illustration of a phylogenetic tree and its corresponding dual graph. a, Each branch point in the phylogenetic tree represents a transmission cluster, and the various branching lengths represent different genetic distances or evolutionary times separating individual nodes. **b,** The vertices and edges in the dual graph correspond to the branches and nodes of the original phylogenetic tree respectively. The size of the node cycle in the dual graph is proportional to the corresponding branch length in the phylogenetic tree.

References

Arık, S. O. and Pfister, T. (2020). Tabnet: Attentive interpretable tabular learning. *arXiv*.

Breiman, L. (2001). Random forests. *Machine learning*, 45(1):5–32.

Chen, T. and Guestrin, C. (2016). Xgboost: A scalable tree boosting system. In *Proceedings of the 22nd acm sigkdd international conference on knowledge discovery and data mining*, pages 785–794.

Dearlove, B. L. and Frost, S. D. (2015). Measuring Asymmetry in Time-Stamped Phylogenies. *PLoS Comput Biol*, 11(7):e1004312.

Drummond, A. J., Rambaut, A., Shapiro, B., and Pybus, O. G. (2005). Bayesian coalescent inference of past population dynamics from molecular sequences. *Mol Biol Evol*, 22(5):1185–1192.

Frost, S. D. W. and Volz, E. M. (2013). Modelling tree shape and structure in viral phylodynamics. *Philosophical transactions of the Royal Society of London. Series B, Biological sciences*, 368(1614):20120208–20120208. 23382430[pmid].

Goldstein, I. H., Bayer, D., Barilar, I., Kizito, B., Matsiri, O., Modongo, C., Zetola, N. M., Niemann, S., Minin, V. M., and Shin, S. S. (2022). Using genetic data to identify transmission risk factors: Statistical assessment and application to tuberculosis transmission. *PLoS Comput Biol*, 18(12):e1010696.

Haykin, S. (1994). *Neural networks: a comprehensive foundation*. Prentice Hall PTR.

Hosmer Jr, D. W., Lemeshow, S., and Sturdivant, R. X. (2013). *Applied logistic regression*, volume 398. John Wiley & Sons.

Kipf, T. N. and Welling, M. (2017). Semi-supervised classification with graph convolutional networks. In *International Conference on Learning Representations (ICLR)*.

Lee, J., Lee, Y., Kim, J., Kosiorek, A., Choi, S., and Teh, Y. W. (2019). Set transformer: A framework for attention-based permutation-invariant neural networks. In *International Conference on Machine Learning*, pages 3744–3753. PMLR.

McKenzie, A. and Steel, M. (2000). Distributions of cherries for two models of trees. *Math Biosci*, 164(1):81–92.

Minin, V. N., Bloomquist, E. W., and Suchard, M. A. (2008). Smooth skyride through a rough skyline: Bayesian coalescent-based inference of population dynamics. *Mol Biol Evol*, 25(7):1459–1471.

Mooers, A. and Heard, S. (1997). Inferring evolutionary process from phylogenetic tree shape. *The Quarterly Review of Biology*, 72(1).

Nee, S., Holmes, E. C., Rambaut, A., and Harvey, P. H. (1995). Inferring population history from molecular phylogenies. *Philos Trans R Soc Lond B Biol Sci*, 349(1327):25–31.

Nee, S., May, R. M., and Harvey, P. H. (1994). The reconstructed evolutionary process. *Philos Trans R Soc Lond B Biol Sci*, 344(1309):305–311.

Oster, A. M., France, A. M., Panneer, N., Bañez Ocfemia, M. C., Campbell, E., Dasgupta, S., Switzer, W. M., Wertheim, J. O., and Hernandez, A. L. (2018). Identifying Clusters of Recent and Rapid HIV Transmission Through Analysis of Molecular Surveillance Data. *J Acquir Immune Defic Syndr*, 79(5):543–550.

Pybus, O. G. and Harvey, P. H. (2000). Testing macro-evolutionary models using incomplete molecular phylogenies. *Proc Biol Sci*, 267(1459):2267–2272.

Pybus, O. G., Rambaut, A., and Harvey, P. H. (2000). An integrated framework for the inference of viral population history from reconstructed genealogies. *Genetics*, 155(3):1429–1437.

Rich, S. N., Richards, V. L., Mavian, C. N., Switzer, W. M., Rife Magalis, B., Poschman, K., Geary, S., Broadway, S. E., Bennett, S. B., Blanton, J., Leitner, T., Boatwright, J. L., Stetten, N. E., Cook, R. L., Spencer, E. C., Salemi, M., and Prosperi, M. (2020). Employing Molecular Phylodynamic Methods to Identify and Forecast HIV Transmission Clusters in Public Health Settings: A Qualitative Study. *Viruses*, 12(9).

Shwartz-Ziv, R. and Armon, A. (2021). Tabular data: Deep learning is not all you need. *Information Fusion*.

Veličković, P., Cucurull, G., Casanova, A., Romero, A., Lio, P., and Bengio, Y. (2018). Graph attention networks. *International Conference on Learning Representations (ICLR)*.

Volz, E. M. and Didelot, X. (2018). Modeling the Growth and Decline of Pathogen Effective Population Size Provides Insight into Epidemic Dynamics and Drivers of Antimicrobial Resistance. *Syst Biol*, 67(4):719–728.

Wakeley, J. and Takahashi, T. (2003). Gene Genealogies When the Sample Size Exceeds the Effective Size of the Population. *Molecular Biology and Evolution*, 20(2):208–213.

Xu, K., Hu, W., Leskovec, J., and Jegelka, S. (2019). How powerful are graph neural networks? *International Conference on Learning Representations (ICLR)*.

Zaheer, M., Kottur, S., Ravanbakhsh, S., Poczos, B., Salakhutdinov, R., and Smola, A. (2017). Deep sets. *Advances in Neural Information Processing Systems*.
